# Time-resolved spectroscopy of a photoactive dinuclear W/Ru complex: spectroscopic evidence for a metastable intermediate with side-on coordinated carbonyl ligand

**DOI:** 10.1039/d6sc00998k

**Published:** 2026-04-17

**Authors:** Jan-Hendrik Borter, Sayan Kangsa Banik, Kristian Kunze, Stephan Kupfer, Dirk Schwarzer, Wolfram W. Seidel

**Affiliations:** a Max Planck Institute for Multidisciplinary Sciences Am Fassberg 11 37077 Göttingen Germany dschwar@mpinat.mpg.de; b Institut für Chemie, Universität Rostock Albert-Einstein-Straße 3a 18059 Rostock Germany wolfram.seidel@uni-rostock.de; c Leibniz-Institut für Katalyse e.V. Albert-Einstein-Straße 29a 18059 Rostock Germany; d Institute of Physical Chemistry, Friedrich Schiller University Jena Helmholtzweg 4 07743 Jena Germany stephan.kupfer@uni-jena.de

## Abstract

The photo-induced dynamics of a redox-active dinuclear W(ii)/Ru(ii) complex, [Tp*W(CO)Br(PyC≡CCH_2_)–Ru(bpy)_2_](PF_6_) (2-PF_6_), is revealed by femtosecond infrared and UV-vis pump-probe spectroscopy in combination with quantum chemical calculations. The use of the mononuclear tungsten alkyne complex [Tp*W(CO)Br(PyC≡CCH_3_)] (1) as a benchmark allowed an in-depth analysis of the excited state kinetics of 2-PF_6_. Excitation of the dinuclear complex at 400 nm produces predominantly a triplet metal-to-ligand charge transfer state localised at the Ru(bpy)_2_ chromophore (^3^MLCT_bpy_) with a lifetime of 6 ps. The following transformation into a tungsten-centered triplet state (^3^MC_W_) is accompanied by significant charge transfer and rearrangement of the W–C–O geometry. Subsequent intersysten crossing back to the ground state on a timescale of 12 ps produces a vibrationally excited molecule with up to 3 quanta in the CO stretching vibration. A minor fraction of 10% of the population reacts to an intermediate exhibiting a lifetime of 140 ps and a CO stretching frequency of 1703 cm^−1^. Our quantum chemical calculations disclose that this species corresponds to an isomer trapped in a metastable state of the S_0_ potential surface, with the CO bound side-on to the W centre.

## Abstract 2

An in-depth investigation of the light-induced dynamics of a redox-active binuclear W(ii)/Ru(ii) complex using femtosecond IR and UV-vis pump-probe spectroscopy allows for the identification of a side-on carbonyl complex intermediate.

## Graphical abstract

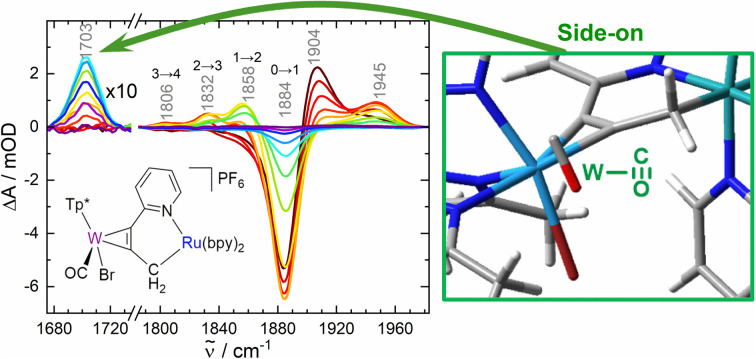

## Introduction

Carbon monoxide complexes are a cornerstone of organometallic chemistry.^1^ The significance of homoleptic compounds as starting materials can hardly be overestimated. The electrophilic character of the metal-bonded C atom opens up synthetic routes to hydrides and carbene complexes. Migratory insertions of CO in metal-carbon bonds as well as cyclizations with coordinated CO being involved are important elementary steps in catalysis. In this context, carbon monoxide is used as a C_1_ feedstock in a variety of processes, which produce value-added base chemicals on mega-tonne scales per annum.^2^ The electronic scope of CO complexes ranges from Ellis' highly reduced carbonyl metallates^3^ to cationic σ-donor CO complexes presented by Willner and Aubke,^4^ which have recently experienced a renaissance by Krossing and coworkers.^5^ In the gas phase, even homoleptic lanthanide and alkaline earth metal carbonyls have been detected by IR photodissociation spectroscopy by Zhou, Li and Frenking.^6^ With regard to the Fischer–Tropsch process, lanthanide and uranium complexes and in particular Nacnac-Mg(i) dimers have been proven to be outstanding in direct C–C coupling of several CO molecules by work of the research groups of Cloke,^7^ Arnold,^8^ Liddle,^9^ Evans^10^ and Jones.^11^

**Chart 1:**
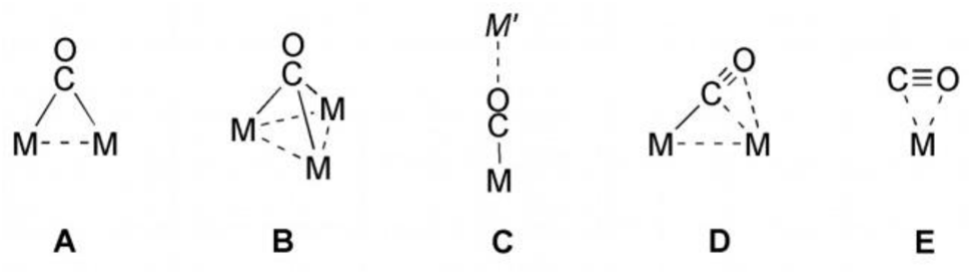
Common bridging modes of CO (A–D) and exclusive side-on coordination mode sought for (E).

The strong preference for end-on C coordination in all these systems based on the frontier orbital composition applies not only to mononuclear but also to polynuclear compounds exhibiting bridging CO. More or less symmetrically carbon bound CO can either reside on an edge (A, Chart 1) or on a surface of a metal cluster (B).^12^ With highly oxophilic metals like lanthanides and actinides a dual C,O-end-on mode is regularly observed (C).^13^ An alternative side-on coordination of CO has so far only been found in bimetallic compounds or metal clusters exhibiting a combined end-on/side-on mode, which was originally called linear semi-bridging by Cotton (D).^14^ A closer look at the structural data in dinuclear complexes of type D underscores the primacy of end-on coordination. Depending on the type of metals linked and their distance from each other, either the M–C bond^15^ or the M–O bond^16^ to the side-on bonded metal can be shorter than the other, with both being longer than 2 Å. In contrast, for the CO ligands in compounds of type D, the terminal M–C bond is particularly short and falls within the range of less than 2 Å. Apparently, the side-on donor effect from the π_CO_ orbitals and the back-bonding into the 
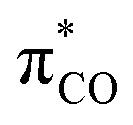
 orbitals by the end-on bound metal constitute a push–pull system, being of crucial relevance for the stability of this motif. Consistently, a mere side-on coordination of the CO ligand to a single metal centre (E) is very elusive and has neither been isolated in substance nor detected spectroscopically. This is in stark contrast to the isoelectronic dinitrogen, of which side-on complexes are well known.^17^ The non-polar nature of N_2_ is an evident reason for this difference, but the exclusive side-on binding is mainly limited to early transition metals, lanthanides, and uranium, and all structurally confirmed N_2_ compounds of this type are dinuclear in the µ–η^2^–η^2^ bonding mode.^18,19^ Significantly, this bonding mode is particularly activating,^19,20^ which makes side-on CO complexes an intriguing intermediate. In addition, linkage isomerism in complexes of the related nitric oxide is subject of spectroscopic investigations and studies by photocrystallographic techniques for some time.^21,22^ Recently, side-on nitrosyl complexes were presented, which are structurally characterized and stable at ambient temperature.^23^

Investigations into the excited state dynamics of carbonyl complexes after UV light irradiation are among the early topics of classical photochemistry seeking a rationale for corresponding synthetic procedures.^24^ The photodissociation of one CO ligand in homoleptic transition metal carbonyls were shown to proceed in a fs time frame, and the rearrangement of the polyhedra in different spin states as well as potential solvent adducts were extensively studied.^25^ Surprisingly, there are no reports on the spectroscopic observation of intermediates with side-on coordination of the CO ligands leaving or remaining in the complex. However, more recently theoretical work describing species with side-on CO coordination at least as minima on the hypersurface has attracted attention.^26^

**Fig. 1:**
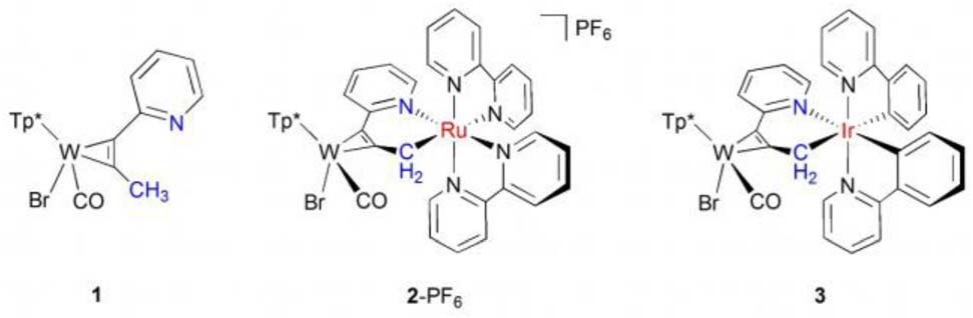
Mono- and dinuclear complexes under study.

As part of our investigations into electron transfer cascades for light-driven charge separation, we have constructed dinuclear complexes, in which the metals are linked by a pyridyl propargyl ligand. The alkyne complex 1 equipped with a pyridine and a methyl substituent (Fig. 1) can act as cyclometalating *C*,*N*-chelate ligand mimicking the binding behaviour of phenylpyridine (ppy), which is used as a cyclometalating monoanionic ligand in many photoactive Ir(iii) compounds. The present W(ii) alkyne complex as a metalla-ligand exhibits additionally a reversible redox process at a potential of about 0 V *vs.* the Fc/Fc^+^ couple, which is capable of reductive quenching of an excited triplet ^3^MLCT transition state at either Ru(ii) or Ir(iii), respectively. The synthesis and spectroscopic characterization of the mononuclear complex 1 and the corresponding dinuclear compounds 2-PF_6_ and 3, including the elucidation of the molecular structures by single crystal XRD and electrochemical studies, are subject of a preceding publication.^27^ The determined redox potentials as well as the significant fluorescence quenching in relation to the parent compounds [(bpy)_2_Ru(ppy)](PF_6_) and [Ir(ppy)_3_] indicate a fast intramolecular electron transfer. To uncover the photodynamic behaviour in depth, transient UV-vis as well as transient IR (TRIR) absorption spectroscopy with the Ru-congener 2-PF_6_ and 1 as mononuclear benchmark were performed in a fs to ps time regime. These experimental investigations are supported by scalar-relativistic time-dependent density functional theory (SR-TDDFT) simulations to elucidate the Franck–Condon photophysics and intersystem crossing (ISC) pathways as well as spectral signatures of photoexcited intermediates of 2-PF_6_. Finally, the synergic synthetic-spectroscopic-theoretical approach allowed to unravel the subsequent excited state relaxation channels associated with electron transfer *vs.* energy transfer. For the first time, we succeeded in spectroscopically detecting a side-on CO complex as an intermediate. Herein, we report on the results of these investigations.

## Results and discussion

The alkyne complex 1 was prepared starting from [Tp*W(CO)_3_] and 2-propinylpyridine. Deprotonation of 1 by KOtBu in the presence of [(bpy)_2_Ru(PPh_3_)Cl]PF_6_ provided 2-PF_6_, which was obtained analytically pure by chromatography and subsequent crystallization. The dark-blue compound exists as an individual diastereomer, which is stable for month under daylight.^27^

To elucidate the photo-induced excited state dynamics in complexes 1 and 2-PF_6_ we applied femtosecond pump-probe spectroscopy. Pump pulse-induced (*λ*_exc_ = 400 nm) absorption changes were monitored in the UV-vis at 350–730 nm using white-light continuum pulses and in the region of the carbonyl stretching frequency (1600–2200 cm^−1^) using IR probe pulses with a bandwidth of about 200 cm^−1^ (details about the experimental setup are presented in a recent publication^28^ and in the SI).

### Mononuclear tungsten complex 1

**Fig. 2:**
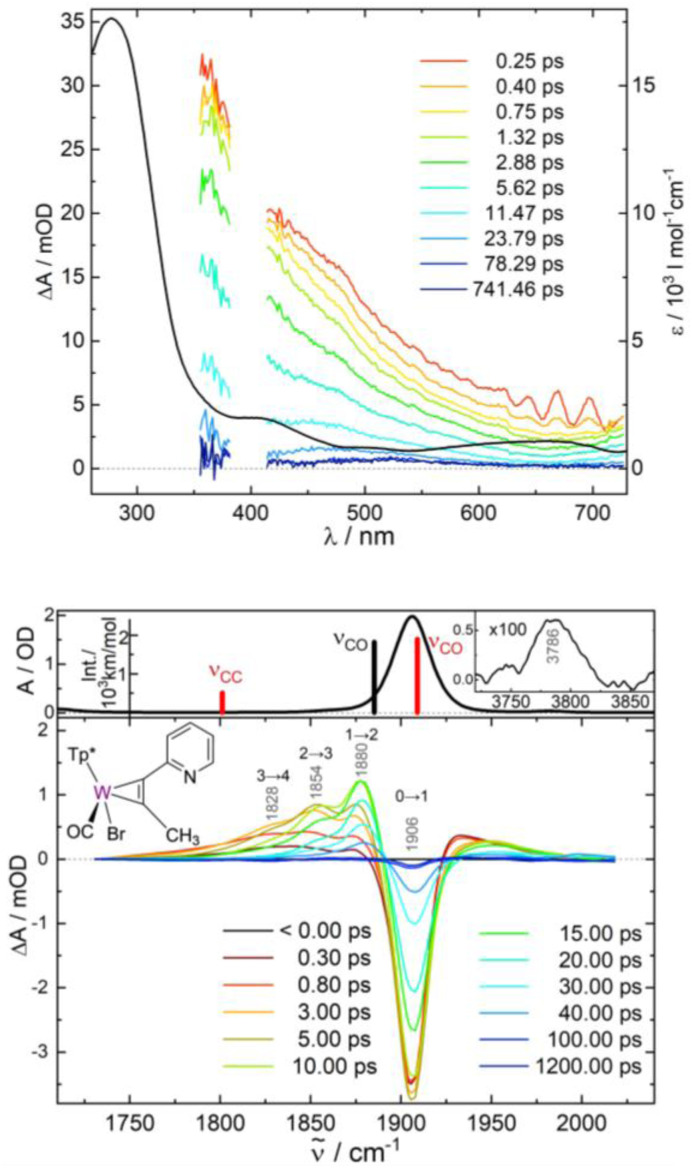
Absorbance changes (Δ*A*) in units of milli optical density (mOD) induced by 400 nm excitation of the tungsten complex 1 in acetonitrile at pump-probe delays as indicated. Upper panel: transients in the UV-vis (regions distorted by the pump pulse are cut out); the black line represents the absorption spectrum (extinction coefficient: right vertical axis). Lower panel: transient IR difference spectra for the CO stretching vibration; the upper insets display the stationary IR spectrum with the fundamental CO transition at *ν*_01_ = 1906 cm^−1^ and the first overtone at *ν*_02_ = 3786 cm^−1^. Stick spectra show calculated frequencies at the B3LYP/def2-SVP level of theory for the ground state (black) and the relaxed T_1_ state of mixed ^3^MC_W_/^3^MLCT character (red).

The absorption spectrum of 1 shows weak bands at 670 and 410 nm, and a stronger one at 280 nm (see black line in Fig. 2, upper panel), which were assigned to W-centred d–d, W-to-pyridine, and W-to-Tp* singlet transitions, respectively.^27^ Pump pulse excitation at 400 nm leads to positive transient absorption over the whole UV-vis range (Fig. 2, upper panel). Its decay is reasonably well fitted by a sum of two exponentials with a dominant contribution exhibiting a time constant of *τ*_2_ = 7 ± 1 ps and a minor one of *τ*_1_ = 0.25 ± 0.1 ps. Transient IR spectra for complex 1 are presented in the lower panel of Fig. 2. The upper insets show the FTIR spectrum of 1 with the fundamental CO stretching vibration at *ν*_01_ = 1906 cm^−1^ and an enlarged image of its overtone absorption at *ν*_02_ = 3786 cm^−1^. Directly after 400 nm excitation the ground state absorption of the CO stretching vibration is bleached and broad almost featureless positive red- and blue-shifted absorptions arise. At pump-probe time delays of 0.3–3.0 ps two bands at 1880 and 1854 cm^−1^ and a shoulder at 1828 cm^−1^ emerge. The frequency spacing between these features, as well as the difference between the 1880 cm^−1^ band and the bleached ground state transition at *ν*_01_ = 1906 cm^−1^, coincide with the anharmonicity of the CO stretching mode determined from the static IR spectrum: 2*ω*_e_*x*_e_ = *ν*_02_ – 2*ν*_01_ = −26 cm^−1^ (*ν*_02_ = 3786 cm^−1^, see Fig. 2). Therefore, we attribute the absorption bands at 1880, 1854, and 1828 cm^−1^ to *ν* = 1 → 2, 2 → 3, and 3 → 4 transitions of the CO stretching mode in the electronic ground state, respectively. Their appearance in less than 0.3 ps indicates excited state lifetimes of less than a few hundred fs, leading to rapid recovery of the ground state. Excited vibrational population in the CO-stretching mode was previously observed in ground state M(CO)_5_(*n*-hexane) (M = Cr, Mo, W) produced by photodissociation of M(CO)_6_.^29^

This assignment to a vibrationally hot electronic ground state species is consistent with the UV-vis transients of Fig. 2. In general, thermal excitation of a molecule leads to broadening of its electronic transitions with absorption being reduced in its centre and enhanced at the low energy side.^30^ The ground state spectrum of 1 is dominated by the relatively strong absorption band at 280 nm. It is conceivable that its thermally induced broadening superimposes any bleaching in the centre of the weak 410 and 670 nm bands resulting in entirely positive absorption changes similar to the UV-vis transients of Fig. 2. Therefore, the dominant decay component of *τ*_2_ = 7 ± 1 ps can be safely assigned to vibrational cooling – a typical value for a large polyatomic molecule in solution.^31,32^ The minor contribution with *τ*_1_ = 0.25 ± 0.1 ps could be due to a short-lived excited state species, most likely the T_1_ state of 1. According to our quantum chemical calculations performed at the density functional level of theory (DFT, B3LYP/def2-svp; see Fig. S3 in the SI for details), this T_1_ state is of triplet metal-centred/metal-to-ligand charge transfer character (*i.e.*^3^MC_W_/^3^MLCT), while the reduced π-back-bonding leads to a slight elongation of the W–CO bond (2.030 Å) with respect to the singlet ground state (1.965 Å). Spectroscopically, the altered electronic structure, *i.e.* in the vicinity of the W–CO motif, results in the blue-shift of the CO stretching mode from 1885 cm^−1^ (S_0_) to 1909 cm^−1^ within T_1_ (see inset in the lower panel of Fig. 2). Assuming a short-lived T_1_, from which fast intersystem crossing (ISC) to the ground state (T_1_–S_0_ gap in ^3^MC_W_ is only 0.25 eV) occurs, would be consistent with the feature seen for the IR transients at 1930 cm^−1^ at early times. The lifetime in the primary populated singlet manifold appears to be below the time resolution of our UV-vis pump-probe spectrometer of about 70 fs.

Population in high *ν*-states of the CO stretching mode evidenced by the IR transients of Fig. 2 indicates a highly non-statistical energy distribution within the molecule after return to the ground state. Note, that based on the frequencies computed from DFT (see Table S2 in the SI) the internal vibrational temperature of 1 after 400 nm photon energy uptake and its statistical distribution over all modes in the ground state would correspond to *T*_exc_ ∼630 K – too low to produce any appreciable population in excited *ν*-states of the CO stretching vibration. A non-statistical distribution with high energy in this mode can be generated by considering the significant MLCT character of the excited state, which shifts electron density away from the metal to the aromatic ligands. This partially depletes antibonding π* orbitals of the carbonyl ligand. Consequently, the CO bond begins to relax from the Franck–Condon region towards the new equilibrium geometry with contracted CO bond. Apparently, the system notably evolves in this direction before it hops back to the ground state surface leaving the CO bond vibrationally excited when now attempting the ground state equilibrium geometry. Non-statistical vibrational populations produced by electronic excitation and ultrafast ground state repopulation have been observed for other tungsten carbonyl complexes before.^29,31^

**Fig. 3:**
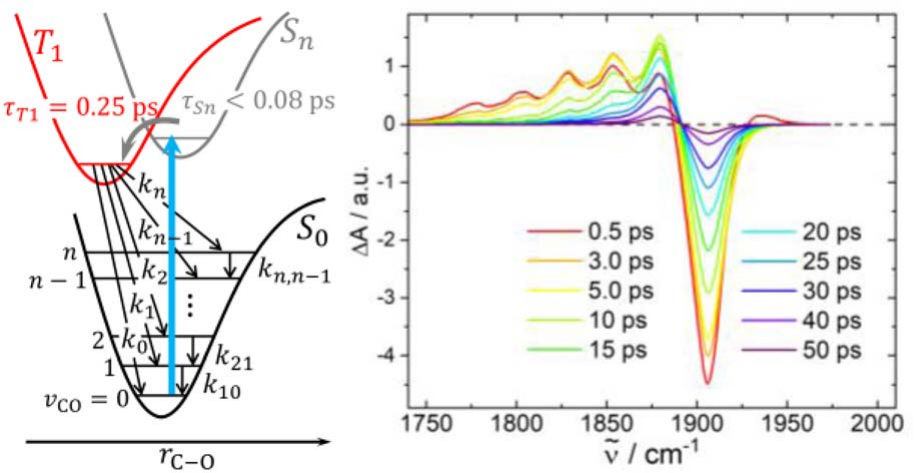
Left panel: kinetic model describing the dynamics of complex 1 after photoexcitation. Rate constants *k*_0_ to *k*_*n*_ determine repopulation of the electronic ground state by intersystem crossing into corresponding *v*-states of the CO stretching mode; *k*_10_ to *k*_*n*,*n*–1_ are vibrational relaxation rate constants. Right panel: simulated spectral evolution (see text).

We fitted the dynamics of the CO stretching vibration by a kinetic model shown in the left panel of Fig. 3. After photo excitation and fast ISC the T_1_ state is populated from which transfer into excited *ν*-states of the CO mode in the ground state occurs. The rate constants *k*_*n*_ are controlling the lifetime of the T_1_ state and the resulting ground state vibrational population distribution, G_*n*_. The ground state relaxation is treated within perturbation theory giving downwards one-quantum transition rate constants of *k*_*n*,*n*–1_ = *n k*_10_; (*n* = 1,2,…)^33^ where *k*_10_ is the relaxation rate constant for a transition *ν* = 1 → 0 of the CO stretching mode. The time-dependent concentrations obtained from the kinetic model are translated into IR difference spectra based on the harmonic approximation giving a linear dependence of the cross section for absorption and stimulated emission on the vibrational quantum number^34^ (the details of this procedure are presented in the SI).

We find good agreement between calculated and measured IR difference spectra when the *k*_*n*_ values are adjusted to produce approximately a Boltzmann distribution at *T*_vib_ = 4500 ± 500 K within the *ν*-states of the CO stretching mode and the vibrational lifetime is set to *τ*_10_ = 1/*k*_10_ = 13 ± 1 ps (see right panel in Fig. 3, details are described in the SI). At early times the modelled spectrum overestimates the modulations caused by populated higher vibrational quantum states of the CO stretching mode. This can be explained by the fact that the model neglects additional shifts caused by off-diagonal coupling to other excited vibrational modes in the molecule, which would lead to additional absorption lines washing out the modulation.^34^ At pump-probe delays >5 ps the agreement between calculated and measured spectra is excellent. The vibrational distribution created after T_1_ → S_0_ ISC corresponds to an average energy in the CO stretching vibration of 〈*E*〉_CO_ ≅ 1560 cm^−1^, indicating that 6% of the applied photon energy flows into this mode when the ground state is repopulated. Since the CO stretching frequency is rather high, its coupling to the solvent is weak. Therefore, the observed energy loss (*τ*_10_ = 13 ps) is likely to be determined by intramolecular vibrational redistribution (IVR) to low frequency modes of the molecule, from where it flows into the solvent. This scenario is consistent with the vibrational cooling time of 7 ps, which was derived from the decay of the UV-vis transients, since the postulated broadening of the 280 nm absorption band is likely caused by other excited ground state vibrations than the CO stretching mode.

In summary, following 400 nm excitation, complex 1 undergoes fast ISC to the T_1_ state with a lifetime of ∼0.25 ps. Subsequent repopulation of the ground state produces a highly non-statistical vibration energy distribution where a significant amount of the photon energy flows into the CO stretching vibration of the molecule. Its transfer to low frequency modes of the molecule happens on a timescale of 13 ps.

### Dinuclear tungsten–ruthenium complex 2-PF_6_

#### UV-vis spectrum

**Fig. 4:**
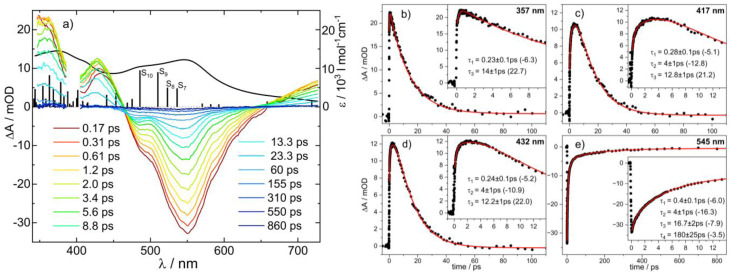
(a): UV-vis transient difference spectra of the W–Ru complex 2-PF_6_ in acetonitrile after 400 nm excitation at pump-probe delays as indicated (regions distorted by the pump pulse are cut out); the black line represents the ground state absorption spectrum (extinction coefficient: right vertical axis); stick spectrum: calculated electronic transitions (TD-B3LYP/def2-svp). (b)–(e): Time traces for selected probe wavelengths with multi-exponential fits (red lines; inserts zoom into the initial phase of the dynamics and show time constants with corresponding amplitudes in brackets).

The UV-vis absorption spectrum of 2-PF_6_ is dominated by ^1^MLCT transitions of the Ru(bpy)_2_-based chromophore (Fig. 4a).^27^ Quantum chemical calculations (TD-B3LYP/def2-svp) enable to assign the underlying electronic transitions to six dipole-allowed singlet ^1^MLCT_bpy_ excitations (into S_7_–S_10_, S_13_ and S_14_) between 536 and 441 nm (2.31–2.81 eV). Notably, S_7_ and S_10_ feature a minor metal-to-metal charge transfer (MMCT) character whereby electron density is partially shifted from the t_2g_ orbitals of Ru to the vacant d orbitals of the W(ii) centre. In addition, and in a similar fashion as observed previously for 1, a weakly dipole-allowed ^1^MC_W_ transition (into S_1_) of the tungsten moiety is predicted at 719 nm (1.72 eV). Further details with respect to the electronic transitions involved in the Franck–Condon region are summarised in the SI (see Fig. S5a and Tables S3 and S4).

#### Pump-probe spectroscopy

As shown in Fig. 4a, excitation at 400 nm leads to immediate bleaching of the strong 550 nm absorption band attributed to Ru-to-bpy ^1^MLCT transitions. Superimposed excited state absorptions produce maxima at 360, 430, and >730 nm. Apart from an instant initial rise, all time traces for the excited state absorptions (Fig. 4b–e) reveal an additional increase on a timescale of ∼0.25 ps. Subsequently, the 360 nm and 730 nm bands decay with a time constant of ∼13 ps, whereas the 430 nm feature exhibits a further significant rise within ∼4 ps before decaying parallel to the other excited state absorptions. The picosecond time constants were found again in the bleach signal, where the 4 ps and 13 ps components are responsible for 48% and 23% of its recovery, respectively. An additional contribution to the ground state recovery of 10% has a time constant of ∼180 ps.

**Fig. 5:**
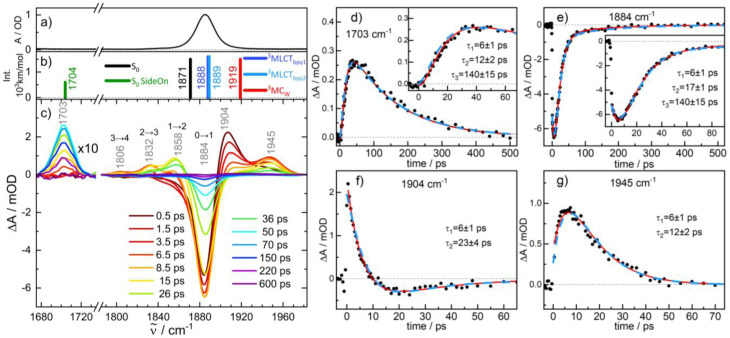
(c) IR transient difference spectra for the CO stretching mode of complex 2-PF_6_ in acetonitrile after 400 nm excitation at pump-probe delays as indicated (the region below 1740 cm^−1^ is 10× enlarged); panels (a) and (b) display the FTIR spectrum and calculated stick spectra for various intermediates. (d)–(g) Time evolution of selected transient IR absorption features with exponential fits (red lines with corresponding time constants); dashed blue lines are time traces from the kinetic model of Fig. 7.

The underlying dynamics are revealed in more detail by the IR transients of Fig. 5 showing well separated absorption features (panel c) and their time evolution (panels d–g), respectively. The photo-induced ground state bleach of the CO fundamental absorption at 1884 cm^−1^ in Fig. 5c is accompanied by two blue-shifted absorptions: a stronger one at 1904 cm^−1^ and a weaker shoulder at 1945 cm^−1^. The following decay of the 1904 cm^−1^ absorption is associated with a further rise of the 1945 cm^−1^ band. Since these processes are exhibiting similar time constants of *τ*_1_ ≅ 6 ps, we tentatively attribute these bands to CO stretching vibrations of the W–Ru complex in two different states (T_2_ and T_1_, respectively) and the observed kinetics to a corresponding transformation of T_2_ into T_1_. A change in the CO bands by 20–60 cm^−1^ to higher frequencies is found in a comparable manner for excited ^3^MLCT states of related Re^I^(CO)_3_ complexes.^35^ Subsequently, the 1945 cm^−1^ feature disappears (*τ*_2_ ≅ 12 ps) and the electronic ground state, G, recovers suggesting a reaction T_1_ → G. Similar to complex 1, ground state recovery leaves the molecule with population in excited states of the CO stretching mode with vibrational transitions at 1884 (*ν* = 0 → 1), 1858 (*ν* = 1 → 2), 1832 (*ν* = 2 → 3), and 1808 cm^−1^ (*ν* = 3 → 4). Note, that the anharmonic shift of −26 cm^−1^ for the CO stretching mode coincides with the value found for 1.

Interestingly, the decay of the T_1_ band also correlates with formation of an absorption feature at 1703 cm^−1^ (rise time ∼12 ps). Clearly, the onset of its rise is slightly delayed with an induction period ∼6 ps suggesting that the growth of the 1703 cm^−1^ band is associated with the decay of T_1_ and not T_2_. The lifetime of this intermediate I of *τ*_3_ ≅ 140 ps agrees with a ∼10% component in the ground state recovery of the *ν* = 0 → 1 transition at 1884 cm^−1^, suggesting that the 1703 cm^−1^ mode of I corresponds to a CO stretching vibration as no other transient absorption features in the range 1650–2200 cm^−1^ were detected.

Note, that we were unable to detect an IR absorption peak at around 1700 cm^−1^ for complex 1. This is consistent with the observation that, during the recovery of the ground state of 1, the bleaching of the *ν* = 0 → 1 transition is always balanced by red-shifted positive absorptions from excited CO vibrational states. However, for 2-PF_6_, these excited state bands are absent during the lifetime of I (see Fig. S7 in the SI).

#### Assignment of the intermediates T_2_ and T_1_

Excitation of the parent complex [Ru(bpy)_3_]^2+^ at around 400 nm is well known to populate a ^3^MLCT state within 40 fs^36^*via* intersystem crossing from the singlet manifold. The ^3^MLCT absorption spectrum shows a shallow, broad absorption in the red and an intense peak at 360 nm.^37^ The latter is attributed to a transition in the reduced bipyridine ligand. The absorption features observed in the initial UV-vis transients displayed in Fig. 4a are strikingly similar to those exhibited by photoexcited [Ru(bpy)_3_]^2+^. This observation leads us to conclude, that after excitation of 2-PF_6_, a Ru-centered ^3^MLCT state is populated within our experimental time resolution. The ^3^MLCT state is therefore also responsible for the CO absorption band of the T_2_ state at 1904 cm^−1^, which appears directly after the pump pulse (Fig. 5c).

**Fig. 6:**
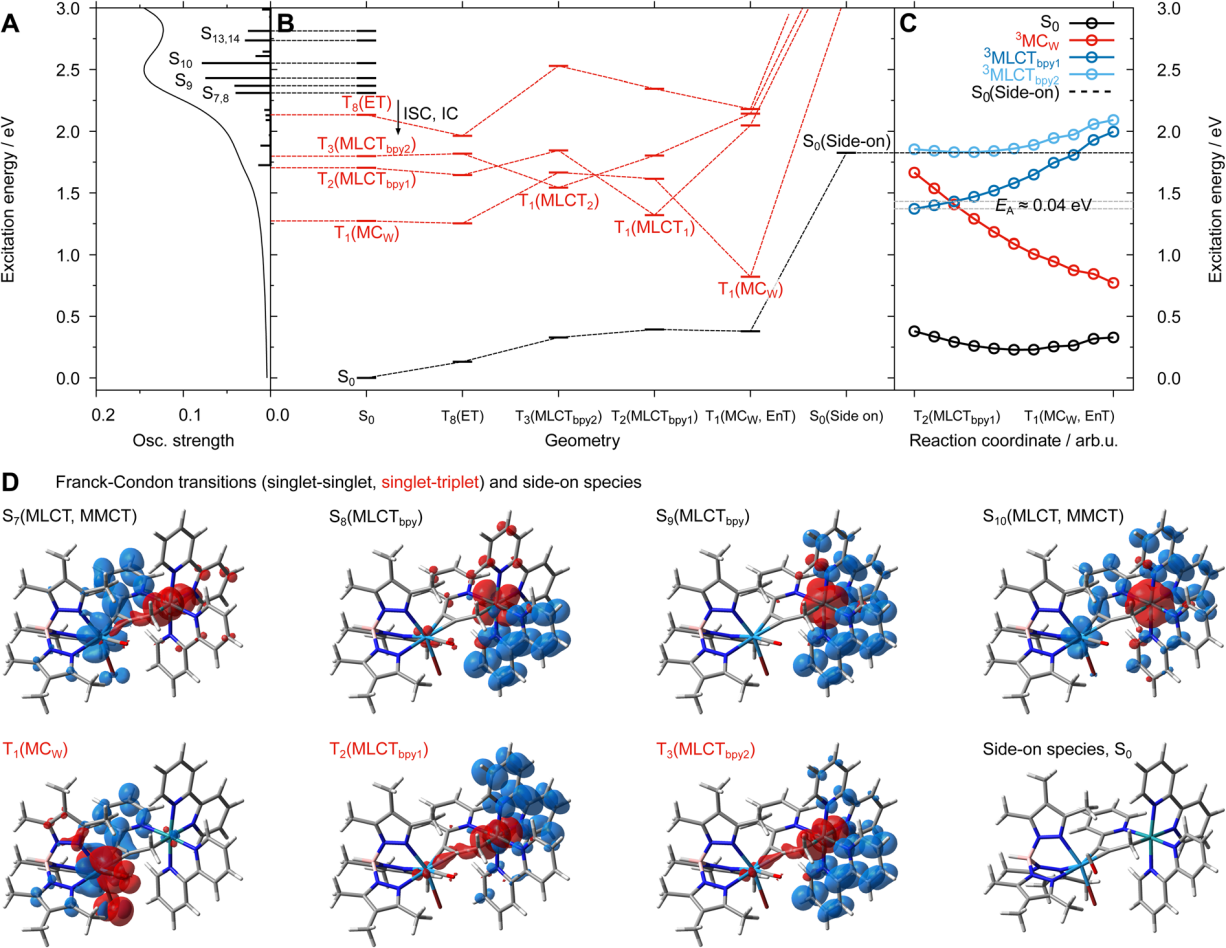
(A): Simulated electronic absorption spectrum of [2]^+^. Prominent dipole-allowed singlet transitions are indicated. (B): Excited-state relaxation scheme involving the initially populated singlet states (in black) and low-lying triplet states (in red) accessible upon intersystem crossing (ISC) and internal conversion (IC) to the ^3^MLCT_bpy_ states (T_3_ and T_2_) and the lowest triplet state of ^3^MC_W_ character as well as the singlet side-on species. (C): Potential energy curves connecting the equilibrated lowest energy ^3^MLCT_bpy1_ state (dark blue) and the ^3^MC_W_ (red) minima; an activation energy of merely 0.04 eV is predicted. (D): Electronic characters of key transitions are visualised by means of charge density difference plot (charge transfer from red to blue) and fully equilibrated side-on species.

This assignment is further supported by quantum chemical calculations within the framework of unrestricted DFT and TDDFT (see details in SI). The first three lowest energy triplet states in the Franck–Condon geometry are a tungsten-centred (^3^MC_W_, T_1_) and two energetically close Ru(bpy)_2_-based metal-to-ligand CT states (^3^MLCT_bpy1_ and ^3^MLCT_bpy2_, T_2_ and T_3_), where charge density is transferred to one or the other bpy ligands (charge density difference plots are shown in Fig. 6).

After geometry optimization the ^3^MLCT_bpy_ states feature almost identical CO stretching frequencies of 1888 and 1889 cm^−1^, which are slightly blue-shifted by ∼18 cm^−1^ with respect to the S_0_ ground state (*ν*_CO_ = 1871 cm^−1^, see Fig. 5b). As the spectroscopic signatures of the two quasi-isoenergetic ^3^MLCT_bpy_ states are almost similar, it is not possible to clarify whether both states coexist or only one dominates. Hence in our discussion we treat the two as a single species.

The relatively small change in CO stretching frequency reflects the fact that in the ^3^MLCT_bpy_ state the electronic and structural environment around the tungsten is quite similar to that in the S_0_ state, causing *e.g.* only a marginal stretching of the W–CO bond from 1.958 Å (S_0_) to 1.968 Å (^3^MLCT_bpy_). The calculated frequency shift agrees well with the experimental S_0_–T_2_ blue shift of 20 cm^−1^, which confirms the assignment of the 1904 cm^−1^ band to the ^3^MLCT_bpy_ state. The appearance of the ^3^MLCT_bpy_ state within the time resolution of our experiment is consistent with scalar-relativistic (SR-)TDDFT simulations yielding sizeable spin–orbit couplings of up to 248 cm^−1^ along these ^1/3^MLCT_bpy_ gateway states (see Table S5), which is within the typical range for 4d transition metal complexes.^38,39^

**Fig. 7:**
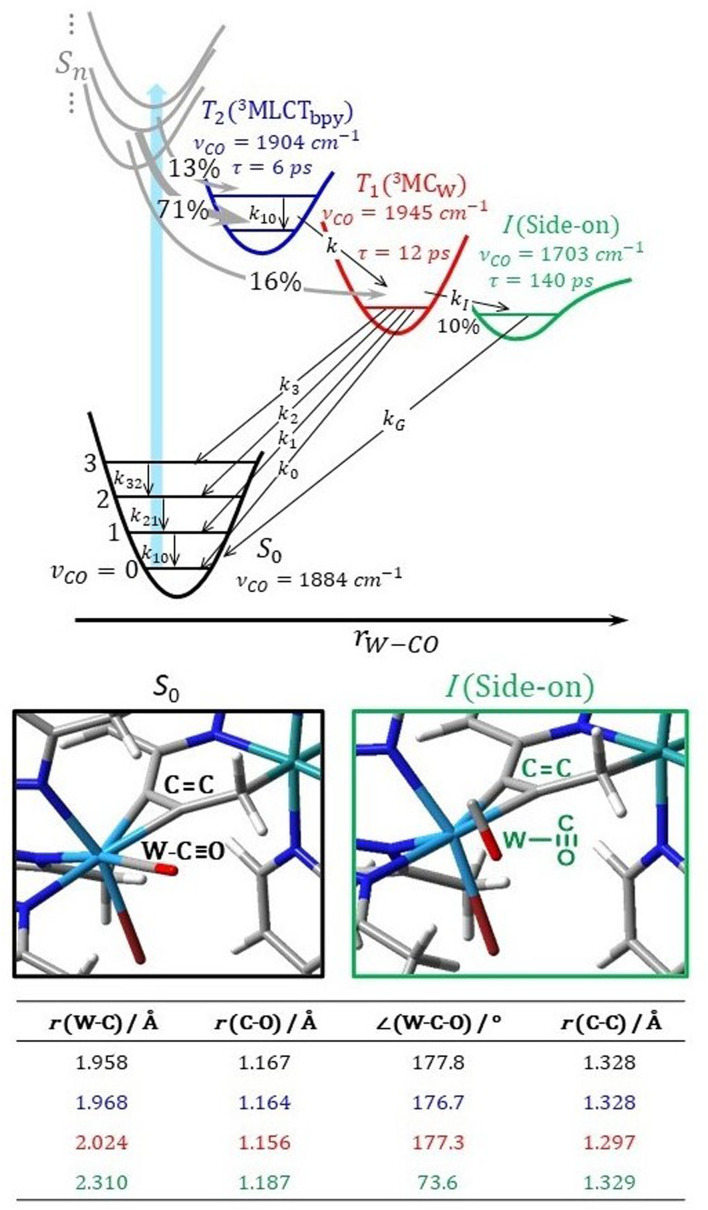
Kinetic model describing the dynamics of complex 2-PF_6_ after photoexcitation (blue arrow) and fast ISC (grey arrows). Within time resolution the states T_2_ and T_1_ are populated in a ratio 0.84 : 0.16; T_2_ transforms into T_1_ with rate constant *k*; T_1_ reacts to the CO side-on complex I (rate constant *k*_1_) or CO stretching mode excited ground state molecules (rate constants *k*_0_ to *k*_3_); *k*_10_ to *k*_32_ are vibrational relaxation rate constants; *k*_G_ determines the lifetime of I. Structural parameters of the W–CO moiety and the propargyl C=C bond length are indicated (black: S_0_, blue: ^3^MLCT_bpy1_, red: ^3^MC_W_, green: side-on); see zoomed in tungsten coordination environment within the singlet ground state and the side-on species. DFT-optimised equilibrium structures are available *via* the free online repository Zenodo (see SI).

By contrast, the ^3^MC_w_ state undergoes major W–CO bond elongation during relaxation to 2.024 Å (see table in Fig. 7), resulting in a CO stretching frequency of 1919 cm^−1^, *i.e.* 48 cm^−1^ higher compared to the S_0_ state. As this compares well with the experimentally observed value for species T_1_ (61 cm^−1^), we attribute the transient CO band at 1945 cm^−1^ to the ^3^MC_w_ state.

The ^3^MLCT_bpy_ → ^3^MC_w_ population transfer suggested by the kinetics of the transient IR bands at 1904 and 1945 cm^−1^ may be viewed as an energy transfer or a dual mutual electron transfer from the ^3^MLCT_bpy_ donor (D) to the ^3^MC_w_ acceptor (A) state, where the corresponding rate constant *k* can be calculated from semi-classical Marcus theory:1
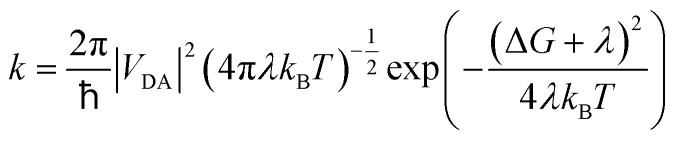


In eqn (1)*V*_DA_ denotes the electronic coupling between D and A at the crossing point of the diabatic potential energy surfaces, *λ* is the reorganization energy, and Δ*G* represents the driving force, *i.e.* the Gibbs free energy, for the process. Notably, recent theoretical investigations have shown that such Marcus-adapted formalism allows to predict not only electron transfer but also the kinetics of (Dexter-type) energy transfer processes.^38–40^ We estimated the rate constant – associated with energy transfer – based on diabatic potentials derived from a linearly interpolated internal coordinate connecting the optimized equilibrium structures of the ^3^MLCT_bpy1_ and ^3^MC_W_ states (resulting in Δ*G* = −0.60 eV and *λ* = 0.76 eV), and applied the minimum splitting method^38,41^ to determine the electronic coupling of *V*_DA_ = 5.5 meV at the crossing point (see the SI for details). The calculated lifetime of *k*^−1^ = 2.4 ps agrees well with the measured time constant of *τ*_1_ = 6 ps associated with the ^3^MLCT_bpy_ → ^3^MC_w_ conversion and confirms the character of this population transfer.

The major decay channel for the ^3^MC_W_ state appears to be ISC to the electronic ground state. We calculate a spin–orbit coupling of 1440 cm^−1^ between the two states consistent with fast relaxation. Notably, the ^3^MC_W_–S_0_ energy gap in [2]^+^ is with 0.44 eV almost twice as big as in 1, which indicates less efficient deactivation in the dinuclear complex.

Taking into account the simulated electronic spectra obtained from TDDFT (see Fig. S5 and S6a in the SI), the reaction cascade ^3^MLCT_bpy_ → ^3^MC_w_ → S_0_ derived from the IR transients is fully consistent with the time-resolved UV-vis data of Fig. 4. The 4 ps rise time observed at probe wavelengths of 430 and 550 nm is close to *τ*_1_ = 6 ps, suggesting its origin in the ^3^MLCT_bpy_ → ^3^MC_w_ transformation. In fact, the calculated cross sections of the ^3^MC_w_ state at these wavelengths are larger than for the ^3^MLCT_bpy_ state. This leads to an increase in excited state absorption at 430 nm and to a significant bleach recovery at 550 nm as this reaction proceeds. Furthermore, the final decay time constant of all UV-vis excited state absorptions is close to *τ*_2_ ≅ 12 ps – in agreement with the ^3^MC_w_ lifetime determined from the IR transients. Finally, the two data sets exhibit a 140–180 ps process, which is responsible for ∼10% of the ground state recovery. The origin of this kinetic component is discussed in the next section.

#### Nature of the intermediate I

The temporal evolution of the 1703 cm^−1^ band for intermediate I shows three main characteristics, *i.e.* (i) delayed response of about 6 ps with respect to the excitation pulse, (ii) subsequent 12 ps rise, which is similar to the lifetime of the T_1_ state, and (iii) final decay with time constant *τ*_3_ ≅ 140 ps. Observation (i) and (ii) suggest a reaction path T_1_ → I. Since *τ*_3_ correlates with a corresponding ground state recovery component of 10%, and no other absorption band was detected in the 1650–2200 cm^−1^ region that could account for the bleached intensity at 1884 cm^−1^, we investigated the possibility that the 1703 cm^−1^ feature is a CO stretching vibration although the observed red shift of 181 cm^−1^ relative to S_0_ appears rather large. Interestingly, similar strong downshifts have been observed for photogenerated nitrosyl complexes, where the NO ligand bound to a transition metal undergoes a transformation from a linear to a side-on geometry (see *e.g.* review Bitterwolf^22^ and references cited therein), suggesting that in I the carbonyl might be bound in a side-on (η^2^) mode.

Our DFT calculations indeed confirm the existence of a metastable isomer of [2]^+^ on the S_0_ potential surface, with the CO bound side-on to the W centre. The energy of this isomer is 1.82 eV above the global minimum, *i.e.* it is accessible by the applied photon energy of *E*_exc_ = 3.10 eV. The complex exhibits a CO stretching frequency of 1704 cm^−1^, which is in excellent agreement with the experimental value of 1703 cm^−1^. According to our calculations is the side-on species notably higher in energy than the ^3^MC_W_ state (Fig. 6B and C) prohibiting a population transfer from the relaxed ^3^MC_W_ to the side-on ground state. This points to a deficiency in the calculations, as the transformation T_1_ → I is clearly evident in the experiment.

We also checked an alternative hypothesis where I corresponds to a fragment generated by cleavage of the W–CO bond in complex [2]^+^. In this scenario the free CO molecule is spectroscopically silent due to its low IR cross section and broad absorption band in solution,^42^ and the 1703 cm^−1^ feature would correspond to the C=C stretching vibration of the alkyne ligand in the fragment. Its complete decay and recovery of the ground state bleach would also imply 100% geminate recombination of the free CO with the fragment on a timescale of 140 ps. In our calculations, however, we find a W–CO bond dissociation energy of 2.31 eV indicating that this reaction path is energetically even more demanding than formation of the side-on complex (at 1.82 eV). Furthermore, in the fragment the C=C stretching vibration is too low in frequency (*ν*_CC_ = 1617 cm^−1^) and not intense enough to account for the observed strength of the 1703 cm^−1^ band (*e.g. ν*_CC_ in Fig. S4). The insertion of CO into the neighbouring bonds was also considered as a potential alternative. While a halocarbonyl ligand is extremely rare and unlikely,^43^ complexes of the C(O)C(R)C(R) building block are known.^44^ However, respective CO vibrations were observed between 1650 and 1670 cm^−1^. Furthermore, our DFT calculations on a putative species of [2]^+^ did not even yield a minimum on the hypersurface. This result is plausible given the 16 valence electron count of the hypothetical species. Thus, we conclude that the intermediate I corresponds to a metastable side-on complex which is formed *via* the non-equilibrated ^3^MC_W_ state with an activated W–CO bond.

#### Kinetic model for the excited state dynamics of 2-PF_6_

The temporal evolution of the IR transients in Fig. 5 and their assignment by our quantum chemical calculations (Fig. 6) suggest an excited state relaxation mechanism, shown in Fig. 7, where the absorption features at 1904, 1945, and 1703 cm^−1^ are attributed to the intermediates T_2(3)_ (^3^MLCT_bpy_), T_1_ (^3^MC_W_), and I (CO side-on isomer in S_0_), respectively.

Photoexcitation into the singlet manifold initially populates states T_2_ and T_1_ by ISC within our time resolution; *i.e.* within few femtoseconds according to our scalar-relativistic TDDFT simulations (Table S7). The branching into the two triplet states is estimated from the extinction coefficient of complexes 1 and 2-PF_6_ at the pump wavelength. These are 2000 and 12 700 L mol cm^−1^, respectively, suggesting that for complex 2-PF_6_ the absorption of a 400 nm photon results in 2000/12 700 = 16% local excitation of the W-alkyne and 84% of the Ru-bpy chromophore. In both cases these local populations quickly (<100 fs) settle in the corresponding lowest triplet states ^3^MC_W_ and ^3^MLCT_bpy_, respectively (grey arrows), giving rise to almost instantaneous absorptions at 1945 (*ν*_CO_ of ^3^MC_W_) and 1904 cm^−1^ (*ν*_CO_ of ^3^MLCT_bpy_). A detailed analysis of the IR transient at a 0.3 ps pump-probe delay (Fig. S1 in the SI) reveals an additional absorption feature 20 cm^−1^ red-shifted from the 1904 cm^−1^ band and covered by the ground-state bleach component. We attribute its origin to a T_2_ population trapped in the first excited vibrational state of the CO stretching mode. The ratio of the *ν* = 0 → 1 to *ν* = 1 → 2 band intensities implies that after ISC, 71% of the total population reaches the T_2_ state in *ν* = 0 and 13% in *ν* = 1 of the CO stretching mode. The population transfer ^3^MLCT_bpy_ → ^3^MC_w_ applies to both T_2_ populations and is described by the rate constant *k* in eqn (1).

The energy of the T_1_ (^3^MC_W_) state in Fig. 7 is near the side-on isomer, which allows a T_1_ → I transformation with rate constant *k*_I_. This process competes with direct relaxation into the vibrationally excited ground state with *n* = 0 to 3 quanta in the CO stretching mode (rate constants *k*_0_ to *k*_3_; higher states are omitted because they were not observed in the IR transients, Fig. 5). Ground state vibrational relaxation is treated in a similar manner as described in the previous section for complex 1. The lifetime of I is so long compared to ground state vibrational relaxation that only the transition into the lowest quantum state *ν*_CO_ = 0 of the ground state *via k*_G_ is considered.

**Fig. 8:**
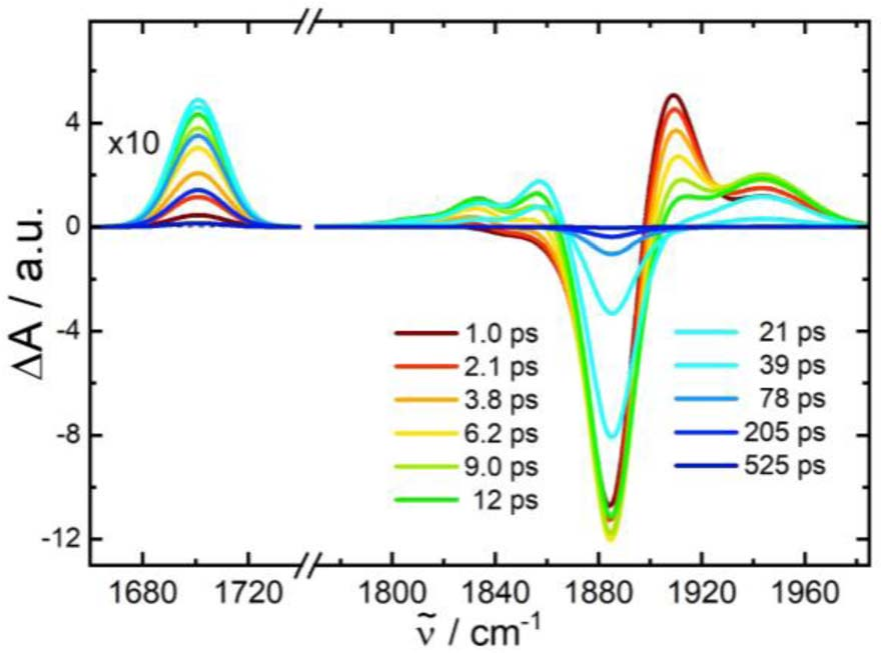
Simulated spectral evolution of IR difference spectra applying the kinetic model of Fig. 7 (for details see text).

Solving the kinetic equations of this model results in time-dependent populations for all states, from which IR difference absorption spectra are calculated using appropriate line shape functions (for details see SI). Fig. 8 shows a fit to the measured IR transients of Fig. 5c yielding lifetimes and branching ratios as noted in Fig. 7 (corresponding rate constants, relative cross sections, and line shape parameters are summarized in Table 1). Moreover, time traces derived from the model are presented in Fig. 5d–g by blue dashed lines. The agreement between the simulated and measured time and spectral evolutions is excellent, supporting the model used.

**Table 1:** Kinetic parameters for the model of Fig. 7 and line shape parameters for fitting the transient IR absorption spectra of complex 2-PF_6_

Species		*σ* a	*v*_0_/cm^−1^^b^	Δ*v*/cm^−1^^c^	Rate constant/ps^−1^
T_2_	*v* = 0	0.76	1904	27	*k* = 0.167, *k*_0_ = 0.033, *k*_1_ = 0.021, *k*_2_ = 0.016, *k*_3_ = 0.0037, *k*_I_ = 0.0083, *k*_G_ = 0.0069, *k*_10_ = 0.16
	*v* = 1	1.52	1884		
T_1_		0.36	1945	35	
I		0.40	1703	21	
*G*_*n*_		(*n* + 1)	*v*_01_ − *n*·2*ω*_e_*x*_e_	—d	

a integrated cross section relative to *σ*_10_ of the S_0_ CO stretching absorption band.

b line center.

c Gaussian line width (FWHM).

d line shape of the stationary FTIR spectrum.

The time-dependent concentrations from this kinetic model (see Fig. S2 in the SI) were also used to simulate UV-vis transients based on the calculated electronic spectra. Although this approach neglects the influence of vibrational excitation on the spectrum the simulated transients (Fig. S6b) reproduce the spectro-temporal evolution of the experimental data (Fig. 4a) surprisingly well.

## Conclusions

In summary, we investigated the excited state dynamics of the mononuclear alkyne complex 1 and the dinuclear complex 2-PF_6_ using femtosecond infrared and UV-vis pump-probe spectroscopy along with quantum chemical calculations. For the reference compound 1, light absorption at 400 nm produces a short-lived (0.25 ps) ^3^MC_W_ state, which repopulates the ground state with up to four quanta in the CO stretching mode, subsequently transferring the excess vibrational energy to the solvent on a 10 ps timescale.

The excited state behaviour of the W–CO moiety changes substantially, when its excitation proceeds indirectly. Photoexcitation of complex 2-PF_6_ primarily generates a Ru(bpy)_2_ localized ^3^MLCT_bpy_ state, which then reacts to a tungsten-centred ^3^MC_W_ state. This involves significant charge redistribution in the complex and a rearrangement of the W–C–O geometry. The calculated lifetime for the ^3^MLCT_bpy_ → ^3^MC_w_ reaction derived from semi-classical Marcus theory of *k*^−1^ = 2.4 ps agrees well with the measured time constant of 6 ps. Compared to 1, is the lifetime of the ^3^MC_W_ state (12 ps) significantly longer due to a larger S_0_–^3^MC_W_ energy gap. The main deactivation channel of the ^3^MC_W_ state in 2-PF_6_ is intersystem crossing to the ground state, similar to the mononuclear complex. Again, we observe a high level of excitation in the CO stretching mode in the S_0_ ground state. In addition, and in contrast to complex 1, we were able to identify a 10% side reaction leading to an intermediate with a CO stretching frequency of 1703 cm^−1^. Our quantum chemical calculations disclose that this species corresponds to an isomer that is trapped in a metastable state on the S_0_ potential surface, with the CO bound side-on to the W centre. To our knowledge, this is the first time that such side-on bound carbonyl complex has been identified experimentally.

## Author contributions

D. S. and W. W. S. conceived the idea behind the manuscript. J.-H. B., S. K. B. and K. K. performed the experimental work and spectroscopic studies. S. K. performed the theoretical calculations. The manuscript was written through the contributions of D. S., S. K. and W. W. S. All authors have approved the final version of the manuscript.

## Conflicts of interest

There are no conflicts to declare.

## Supplementary Material

SC-017-D6SC00998K-s001

## Data Availability

The data supporting this article have been included in the main manuscript as well as in the supplementary information (SI). Furthermore, all optimized structures and high-resolution images are available *via* the free online repository Zenodo (DOI: 10.5281/zenodo.15083786). Supplementary information: experimental details of transient absorption spectroscopy, kinetic models and comprehensive computational results. See DOI: https://doi.org/10.1039/d6sc00998k.
