# Supplementary material for: Time-resolved spectroscopy of a photoactive dinuclear W/Ru complex: spectroscopic evidence for a metastable intermediate with side-on coordinated carbonyl ligand
Source: Chem Sci. 2026 Apr 17;17(21):10600–10. doi: 10.1039/d6sc00998k (PMC13088430; doi:10.1039/d6sc00998k)
Supplement: SC-017-D6SC00998K-s001 [file SC-017-D6SC00998K-s001.pdf]

Supplementary Information (SI) for Chemical Science.
This journal is © The Royal Society of Chemistry 2026

Supplementary Information

Time-resolved spectroscopy of a photoactive dinuclear W/Ru complex: Spectroscopic evidence for a metastable intermediate with *side-on* coordinated carbonyl ligand

Jan-Hendrik Borter,[a] Sayan Kangsa Banik,[a] Kristian Kunze,[b,c] Stephan Kupfer,*[d] Dirk Schwarzer,*[a] and Wolfram W. Seidel*[b,c]

[a] *Max Planck Institute for Multidisciplinary Sciences, Am Fassberg 11, 37077 Göttingen, Germany. E-mail: dschwar@mpinat.mpg.de*
[b] *Institut für Chemie, Universität Rostock, Albert-Einstein-Straße 3a, 18059 Rostock, Germany. E-mail: wolfram.seidel@uni-rostock.de*
[c] *Leibniz-Institut für Katalyse e.V., Albert-Einstein-Straße 29a, 18059 Rostock, Germany.*
[d] *Institute of Physical Chemistry, Friedrich Schiller University Jena, Helmholtzweg 4, 07743 Jena, Germany. E-mail: stephan.kupfer@uni-jena. de*

1. Experimental section

1.1. Transient absorption spectroscopy

The laser spectrometer to record transient UV-vis and mid-IR spectra is described in detail by Julia Franz et a.[1] UV-vis and mid-IR experiments were performed with sample concentrations of <1 mM in a quartz glass cell (2 mm path length) and a stainless-steel cell equipped with CaF_2 windows (0.5 mm path length), respectively. Magnetic stirrers were employed to avoid accumulation of photoproducts in the laser focus. Samples were excited with pump energies of 2 μJ focused to a diameter of 0.2 mm.

1.2. Kinetic model for the IR transient absorption spectra of complex 1

The kinetic model shown in Figure 3 (left panel) of the main paper results in a set of coupled differential equations for the time-dependent concentrations in the CO stretching v-states of the ground and the triplet state, G_n and T_1, respectively (short-lived excited singlet and triplet states are omitted; only the vibrational ground state of the T_1 is considered)

$$\frac{dT_1}{dt} = -\frac{T_1}{\tau_{T1}}$$

$$\frac{dG_0}{dt} = k_0 T_1 + k_{10} G_1 \qquad \text{(S1)}$$

$$\frac{dG_n}{dt} = k_n T_1 + (n+1) k_{10} G_{n+1} - n\, k_{10} G_n \quad ; \text{for } n = 1, 2, \ldots$$

In Eqs. S1 the lifetime of T_1 is given by $\tau_{T1} = 1/\sum k_n$ where k_n represents the rate constant for repopulating the ground vibrational state n. Within perturbation theory bilinear coupling of the CO

oscillator to a thermal bath results in one quantum allowed transitions ($\Delta v = \pm 1$) where the downward rate constants $n \to n-1$ are proportional to the vibrational quantum number[6, 7], i.e.

$$k_{n,n-1} = n \cdot k_{10}\ ;(n = 1, 2,\ldots) \qquad \text{(S2)}$$

Moreover, upward and downward rate constants are coupled by detailed balance

$$\frac{k_{n-1,n}}{k_{n,n-1}} = \exp\left(-\frac{(E_n - E_{n-1})}{k\,T}\right) \qquad \text{(S3)}$$

where $E_n - E_{n-1}$ corresponds to the energy difference between the vibrational states, and k and T are Boltzmann constant and temperature, respectively. In general, the detailed balance condition ensures that the population approaches the correct equilibrium distribution for $t \to \infty$. In the case of the CO stretching vibration the upward process $n-1 \to 1$ can be neglected because at room temperature the Boltzmann factor in Eq. (S3) is only $\exp\left(-\frac{hc\,v_{01}}{k\,T}\right) = 10^{-4}$. Eq. (S2) allows to describe the vibrational relaxation cascade and the associated emerging time-dependent population distribution $G_n(t)$ in the ground state by a single parameter, k_{10}.

The rate constants k_n were adjusted to result in a T$_1$ lifetime of $\tau_{T1} = 0.25\ ps$ (consistent with the fast decay component seen in the UV-vis transients of **1**) and, at the same time, to create a truncated Boltzmann distribution within the CO stretching vibrational manifold of the ground state, i.e.

$$k_n \propto \left[\exp\left(-n \cdot \frac{hc\,v_{01}}{k\,T_{vib}}\right) - \exp\left(-(n_{max} + 1) \cdot \frac{hc\,v_{01}}{k\,T_{vib}}\right)\right] \qquad \text{(S4)}$$

where T_{vib} corresponds to an adjustable vibrational temperature and n_{max} is the highest v-state taken into account for ground state repopulation. Since the IR transients in Figure 2 fade out below 1750 cm^{-1} a value of $n_{max} = 5$ was chosen and $k_n = 0$ for $n > n_{max}$.

For calculating spectra we applied the harmonic approximation giving a linear dependence of the cross section for absorption and stimulated emission on the vibrational quantum number[8]. Hence, the ground state absorption spectrum is represented by

$$A_G(v,t) = \sum_{n=0}^{n_{max}} (G_n(t) - G_{n+1}(t))(n+1)\sigma_{01} \cdot L_G(v - v_{01} + n \cdot 2\omega_e x_e) \qquad \text{(S5)}$$

Where $G_n(t) - G_{n+1}(t)$ is the population difference between adjacent vibrational states n and $n+1$, σ_{01} is the absorption cross section for the $v = 0 \to 1$ transition, $L_G(v)$ is a line shape function, and $2\omega_e x_e$ is the anharmonicity of the CO stretching vibration. Difference spectra were calculated by adding the time-dependent absorption of the triplet state

$$A_{T1}(v,t) = T_1(t)\ \sigma_{T1} \cdot L_{T1}(v - v_{T1}) \qquad \text{(S6)}$$

(with corresponding population $T_1(t)$ in the $v = 0$ state, cross section σ_{T1}, and line shape function $L_{T1}(v)$ of the $v = 0 \to 1$ transition) and subtracting the ground state room temperature spectrum $A_G(v,300K)$ when the relaxation is completed (eq.(S5) with $G_0 = 1$ and all other populations zero), i.e.

$$\Delta A(v,t) = A_G(v,t) + A_{T1}(v,t) - A_G(v,300K)\ . \qquad \text{(S7)}$$

In Eq. (S5) the line shape function for all ground state transitions was assumed to be identical to the FTIR spectral line shape of the fundamental mode (upper panel in Fig. 2a of the main text). For the T_1 state a Gaussian line shape of the form

$$g(\nu) = \frac{2\,\sigma}{\Delta\nu}\sqrt{\frac{\ln(2)}{\pi}}\exp\left[-4\ln(2)\left(\frac{\nu-\nu_0}{\Delta\nu}\right)^2\right] \tag{S8}$$

was employed, where σ is the integrated cross section, $\Delta\nu$ the full width at half maximum (FWHM) and ν_0 the line center of the transition.

Using the initial relative populations $(T_1)_{t=0} = 1$ and $(G_n)_{t=0} = 0$ and adjustable values for k_{10}, T_{vib}, and the line shape parameters, the experimental transient difference spectra were fitted. The result shown in Figure 3 (right panel) of the main text was obtained with $k_{10} = 0.077\,ps^{-1}$ and $T_{vib} = 4500 \pm 500\,K$. The complete set of fit parameters is summarized in Table S1.

Table S1. Line shape and kinetic parameters for fitting the transient IR absorption spectra of complex **1**.

Species	σ [a]	ν_0/cm^{-1} [b]	$\Delta\nu$/cm^{-1} [c]	Rate constant/ps^{-1}
T_1	1	1925	30	$k_0 = 2.088$, $k_1 = 1.035$, $k_2 = 0.504$, $k_3 = 0.237$, $k_4 = 0.102$, $k_5 = 0.034$, $k_{10} = 0.077$
G_n	$(n+1)$	$\nu_{01} - n \cdot 2\omega_e x_e$	— [d]	

[a] integrated cross section relative to σ_{01} of the ground state CO stretching absorption band;
[b] line center; [c] Gaussian line width (FWHM); [d] line shape of the stationary FTIR spectrum.

1.3. Decomposing the early-time IR transients of the W-Ru complex 2-PF_6 into excited-state absorption and ground-state bleach

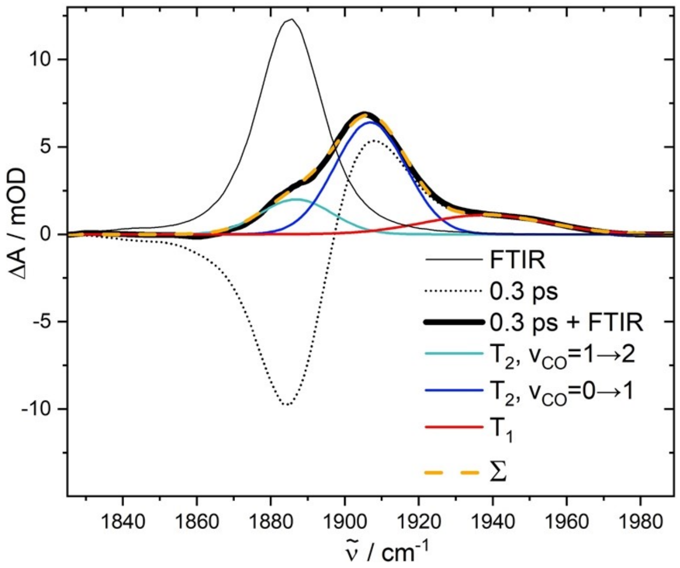

Figure S1. The pure excited state absorption (thick black line) in the 0.3 ps pump-probe delay transient IR spectrum is obtained by removing the ground state bleach component. This is done by adding the scaled FTIR spectrum (thin black line) to the 0.3 ps transient (dotted line), with the scaling factor chosen so that the negative absorption at the red edge of the spectrum just disappears. The resulting excited state absorption is fitted by a sum of three Gaussians (dashed yellow line) centred at 1884, 1904, and 1945 cm^{-1}. The first two are attributed to the v = 1→2 (light blue) and v = 0→1 (dark blue) transitions of the CO stretching vibration in the T_2 state, the latter (red) corresponds to the fundamental CO transition in the T_1 state of the W-Ru complex **2**-PF_6.

1.4. Kinetic model for the IR transient absorption spectra of 2-PF_6

The kinetic model shown in Figure 6 of the main paper results in the following set of coupled differential equations for the time-dependent concentrations of the T_2 state in $v_{CO} = 1$ and 2, $T_{2,1}$ and $T_{2,0}$, respectively, as well as T_1, I, and the excited CO stretching v-states of the ground state (neglecting all short-lived precursor states as e.g. S_n):

$$\frac{dT_{2,1}}{dt} = -(k_{ET} + k_{10})T_{2,1}$$

$$\frac{dT_{2,0}}{dt} = -k_{ET}T_{2,0} + k_{10}T_{2,1}$$

$$\frac{dT_1}{dt} = k_{ET}(T_{2,1} + T_{2,0}) - k_I T_1 - \left(\sum_{n=0}^{3} k_n\right) T_1 \qquad \text{(S9)}$$

$$\frac{dI}{dt} = k_I T_1 - k_G I$$

$$\frac{dG_0}{dt} = k_0 T_1 + k_G I + k_{10} G_1$$

$$\frac{dG_n}{dt} = k_n T_1 + (n+1)k_{10}G_{n+1} - n\,k_{10}G_n \text{ ; for } n = 1, 2, 3$$

The resultant time-dependent concentrations are used to calculate the IR difference absorption spectrum

$$\Delta A(\nu,t) = A_G(\nu,t) + A_{T2}(\nu,t) + A_{T1}(\nu,t) + A_I(\nu,t) - A_G(\nu,300K) \quad \text{(S10)}$$

where

$$A_G(\nu,t) = \sum_{n=0}^{3}\left(G_n(t) - G_{n+1}(t)\right)(n+1)\sigma_{01}\cdot L_G(\nu - \nu_{01} + n\cdot 2\omega_e x_e) \quad \text{(S11)}$$

$$A_{T2}(\nu,t) = \left(T_{2,1}(t) - T_{2,0}(t)\right)\sigma_{T2}\cdot L_{T2}(\nu - \nu_{T21}) + T_{2,1}(t)\cdot 2\sigma_{T2}\cdot L_{T2}(\nu - \nu_{T20}) \quad \text{(S12)}$$

$$A_{T1}(\nu,t) = T_1(t)\,\sigma_{T1}\cdot L_{T1}(\nu - \nu_{T1}) \quad \text{(S13)}$$

$$A_I(\nu,t) = I(t)\,\sigma_I\cdot L_I(\nu - \nu_I) \quad \text{(S14)}$$

and $A_G(\nu,300K)$ corresponds to the ground state room temperature spectrum when the relaxation

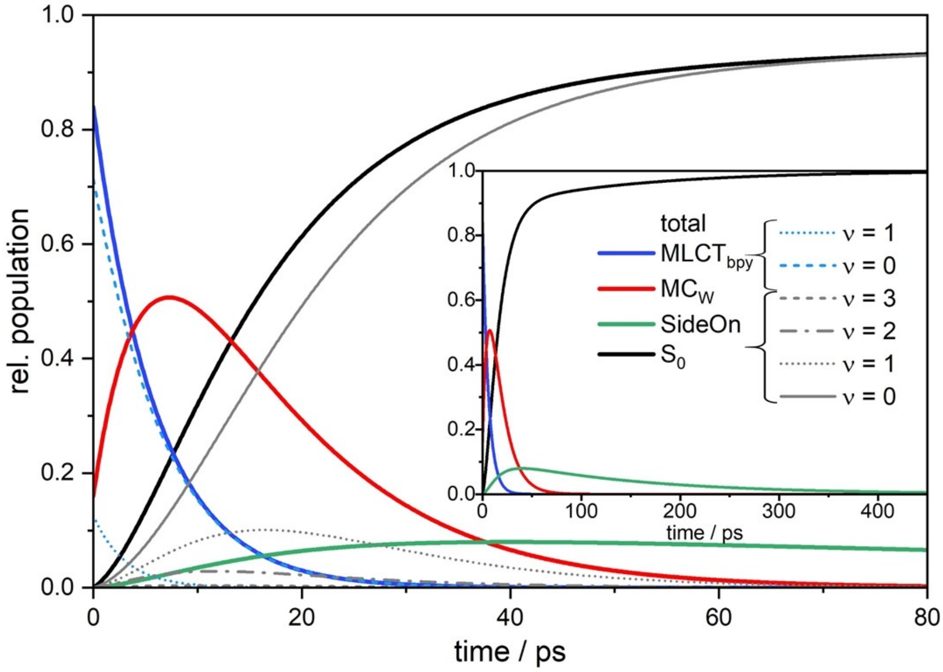

Figure S2. Time-dependent populations for the photo-excited complex **2**-PF_6 resulting from the kinetic model of Figure 7 and rate constants of Table 1 of the main paper.

is completed and all the population ends in G_0. For the line shape function L_G of all ground state transitions we used the FTIR spectral line shape of the stationary absorption spectrum (Figure 5 of the main text). For L_{T2}, L_{T1}, and L_I Gaussian line shapes (Eq. (S8)) were employed. The simulated IR transients of Figure 7 were obtained with a set of parameters presented in Table 1. Corresponding time-dependent concentrations are shown in Figure S3.

1.5. Calculation of UV-vis transient absorption spectra for 2-PF_6

The simulation of time-dependent UV-vis transients is based on the ground state and excited state spectra $S_i(\lambda)$ derived from TDDFT calculations (see Figure S5) and the kinetic model from the previous section, which provides time-dependent populations for all relevant states. In contrast to the IR spectra, for the calculation of the UV-vis transients we neglect the influence of vibrational excitation in the CO stretching or any other mode on the electronic spectrum. We also neglect the possibility of vibronic excitation in the absorption spectra. Then the UV-vis transient difference spectrum is given by

$$\Delta A(\lambda,t) = T_2(t) \cdot S_{T2}(\lambda) + T_1(t) \cdot S_{T1}(\lambda) + I(t) \cdot S_I(\lambda) + (1 - G(t)) \cdot S_G(\lambda) \qquad \text{(S15)}$$

Where $T_2(t) = \sum_{n=0}^{2} T_{2,n}(t)$, $T_1(t)$, $I(t)$, and $G(t) = \sum_{n=0}^{3} G_n(t)$ are the time-dependent populations in each electronic state. The calculated UV-vis transients are shown in Figure S6.

2. Quantum chemistry

2.1. Computational details

All quantum chemical calculations assessing the structural and electronic properties of complexes **1** and [**2**]$^+$ were carried out using the Gaussian 16 program.[2] Density functional theory (DFT) was employed to obtain the fully relaxed singlet ground state (S_0) and the lowest triplet state (T_1) of the two complexes. Furthermore, two possible *side-on* species of [**2**]$^+$ were investigated within singlet and triplet multiplicity, i.e., with the CO ligand being coordinated via one of its π_{CO}/π^*_{CO} orbitals. The triplet species of the *side-on* complex was found to be dissociative and was not investigated further. Finally, the possible W-CO bond breakage in [**2**]$^+$ was investigated (singlet and triplet) applying the B3LYP XC functional[3] in combination with the all-electron def2-SVP basis set.[4] Additionally, dispersion correction was included using the GD3 model with Becke-Johnson damping.[5] Implicit solvent effects (acetonitrile, ε = 35.688) were taken into account by the polarizable continuum model (PCM) using the equilibrium procedure of the SMD solvation model.[6] The vibrational frequency calculations showed that all obtained geometries are minima of the potential energy surface. In order to account for the approximation treatment of electron correlation and anharmonicity, all simulated vibrational frequencies were scaled by a factor of 0.95.[7] All fully optimised equilibrium structures are freely available via the Zenodo online repository.[8]

Subsequently, time-dependent DFT (TDDFT) was applied to assess the singlet and triplet excited states properties (i.e. electronic characters, energies and oscillator strengths) of **1** and [**2**]$^+$, exclusively. Therefore, the same computational setup was employed as in the initial ground state calculations. Several studies on structurally related photoactive Ru(II)-based transition metal complexes showed that such computational protocol – combining hybrid functionals with a medium amount of exact-exchange and double-ζ basis sets – enables an accurate prediction of ground and excited states properties with respect to experimental data, e. g. UV-vis absorption, resonance Raman spectra, (spectro-)electrochemical, transient absorption and electron transfer properties.[9] A balanced description of excited states featuring, i. e., metal-to-ligand charge transfer (MLCT), ligand-to-metal charge transfer (LMCT), ligand-to-ligand charge transfer (LLCT), intra-ligand charge transfer (ILCT) and metal-to-metal charge transfer (MMCT) in case of binuclear coordination compounds such as the present W/Ru complex as well as intra-ligand (IL) and metal-centred (MC) character, as provided by the present computational setup, is essential to evaluate the rich photophysics of transition metal complexes. The excited state properties within the Franck-Condon point (S_0 equilibrium structure of [**2**]$^+$) were evaluated by means of the non-equilibrium procedure of solvation to estimate the initial vertical absorption energies. The 100 lowest energy singlet-singlet excitations were obtained to model the electronic absorption spectra of [**2**]$^+$. In addition, the 100 lowest energy (dipole-forbidden) singlet-triplet transitions were evaluated in order to address the availability of prominent triplet states involved in the subsequent intersystem crossing (ISC) and excited state relaxation pathways. Furthermore, scalar-relativistic (SR-)TDDFT calculations were performed utilizing Orca 5.0.37[10] with scalar relativistic Douglas Kroll Hess Hamiltonian of 2nd-order to assess prominent pathways for intersystem crossing within the S_0 structure, while the analogue computational protocol was applied as in the Gaussian 16 simulations (e.g., B3LYP/def2-svp and corresponding autoaux auxiliary basis set, SMD: acetonitrile). The 20 lowest singlet-singlet and singlet-triplet excitations were obtained, while spin-orbit couplings (SOCs) between these states and the singlet ground state were obtained at the SR-TDDFT level of theory.

Subsequently, to quantify the rate of intersystem crossing (ISC), the simplified formula of rate constant with Fourier transformed Lorentzian dephasing[11] was utilized, which was recently applied in the context of ISC in a Cr(III) spin-flip emitter.[12] For the energy downhill $S_i \rightarrow T_j$ rate constant, the formula is shown as below

$$k_{ISC\downarrow} = \frac{2\gamma}{\hbar(\Delta E_{ij}^2 + \gamma^2)} |SOC|^2 \tag{S16}$$

where the γ represents the half width at half maximum (HWHM) of the simulated UV-vis spectrum, ΔE_{ij} is the vertical energy difference at FC region, SOC denotes the spin-orbit coupling term and ħ is the reduced Planck constant. In a similar manner, when the $S_i \rightarrow T_j$ non-radiative transition is an energy-uphill transition, the correction is added:

$$k_{ISC\uparrow} = \frac{2\gamma}{\hbar(\Delta E_{ij}^2 + \gamma^2)} |SOC|^2 exp\left(-\frac{\Delta E_{ij}}{k_B T}\right) \tag{S17}$$

where k_B is the Boltzmann constant and T is the temperature (T = 293.15 K).

The lowest three triplet excited states (singlet-triplet excitations; of T_1: ^3MC_W, T_2: $^3MLCT_{bpy1}$, T_3: $^3MLCT_{bpy2}$) were relaxed at the TDDFT level of theory. All three triplet states relax to the lowest energy triplet state in their respective equilibrium structure and were optimised subsequently using (unrestricted) DFT. In case of **1**, only the lowest three singlet-triplet states within the equilibrated ^3MC_W (T_1) were assessed by TDDFT.

In addition, the time-resolved IR (TRIR) (**1** and [**2**]$^+$) and transient absorption (TA) UV-vis spectra (only [**2**]$^+$) were modelled within the three optimised triplet (ground) states, i.e., of the ^3MC_W and the two $^3MLCT_{bpy}$ states as well as of the singlet *side-on* species (only shown for lowest energy *side-on* complex). In case of TRIR, the excited state absorption was modelled by means vibrational normal modes as obtained within the relaxed triplet states, respectively. Ground state bleach was estimated based on the vibrational normal modes of the singlet ground state (within the Franck-Condon point). In case of TA-UV-vis, an analogue procedure was applied, while the excited state absorption was modelled by the lowest 100 spin and dipole-allowed triplet-triplet transitions as obtained within the previously optimized T_1 equilibria and ground state bleach via the singlet-singlet transitions as obtained within the relaxed singlet ground state. In all transient spectra a 1:1 population of S_0 and T_1, was assumed. In case of the side-on complex, a similar procedure was performed however the ESA was modelled by the singlet species rather than by the respective (dissociative) triplet analogue.

Finally, to access the kinetics of photoinduced electron transfer (ET) processes in [**2**]$^+$, semi-classical Marcus theory was applied. According to Marcus theory, ET processes occur along the parabolic diabatic potential energy curves (PECs) of the electron donor state (D; *i.e.* $^3MLCT_{bpy1}$) and the acceptor state (A; *i.e.* ^3MC_W) along the reaction coordinate R_{ET}. Thereby, thermal fluctuations of the solvent may lead to structural distortions within the donor state that provide sufficient electronic coupling between D and A to allow a population transfer between these electronic states. Herein, the rate constant, k, for such an ET is given within the semi-classical Marcus-picture by:

$$k = \frac{2\pi}{\hbar}|V_{DA}|^2(4\pi\lambda k_B T)^{-\frac{1}{2}} \exp\left(-\frac{(\Delta G + \lambda)^2}{4\lambda k_B T}\right), \tag{S18}$$

where V_{DA} denotes electronic coupling between the D and A states at the crossing point of the diabatic PECs, λ is the reorganization energy, ΔG represents the driving force, *i.e.* the Gibbs free energy, for the ET reaction, k_B is Boltzmann constant, T is absolute temperature.

In case of [**2**]$^+$, D and A states of interest are of triplet multiplicity. The ET kinetics were described along a linear-interpolated internal coordinate (LIIC) connecting the optimized equilibrium structures of the $^3MLCT_{bpy1}$ (D) and the ^3MC_W (A) states. The diabatic PECs for D and A were constructed along the LIIC (denoted R_{ET}) by means of TDDFT single-point calculations. The electronic coupling $V_{DA}(R_{ET})$ was obtained by a unitary transformation of the adiabatic states, $V_1^{ad}(R_{ET})$ and $V_2^{ad}(R_{ET})$, to the respective diabatic states, ($V_D(R_{ET})$ and $V_A(R_{ET})$:

$$\begin{pmatrix} V_A(R_{ET}) & V_{AD}(R_{ET}) \\ V_{DA}(R_{ET}) & V_D(R_{ET}) \end{pmatrix} = U^{-1} \begin{pmatrix} V_1^{ad}(R_{ET}) & 0 \\ 0 & V_2^{ad}(R_{ET}) \end{pmatrix} U \quad , \qquad \text{(S19)}$$

in which U is a general Unitary matrix, *i.e.,* $\begin{pmatrix} \cos\theta & \sin\theta \\ -\sin\theta & \cos\theta \end{pmatrix}$. The electronic coupling is then defined as:

$$V_{DA} = \frac{1}{2}\left|V_2^{ad} - V_1^{ad}\right|\sin(2\theta) \quad . \qquad \text{(S20)}$$

At the crossing point of the diabatic PEC ($V_A = V_D$) a mixing angle of $\theta = \pi/4$ is obtained and S20 simplifies to:

$$V_{DA} = \frac{1}{2}\left|V_2^{ad} - V_1^{ad}\right|_{min} \quad , \qquad \text{(S21)}$$

which is known as the minimum splitting method. This computational protocol was recently introduced and successfully applied to assess the intramolecular electron transfer kinetics in the frame of solar energy conversion and storage.[9b,9c,13]

2.2. Computational results

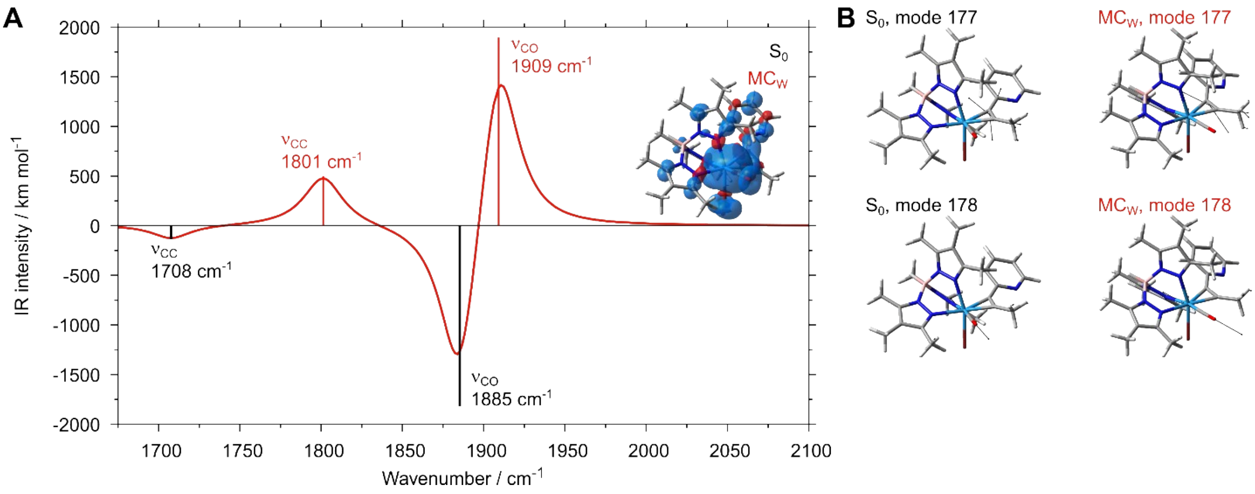

Figure S3. A: Simulated transient IR (TRIR) spectra of the tungsten complex **1** obtained at the B3LYP/def2-SVP level of theory with contributions of excited-state absorption given by vibrational normal modes within the fully relaxed T_1 state of mixed $^3MC_W/^3MLCT$ character (red; see spin density as inset). Key vibrations are indicated. **B**: Respective equilibrium structures and normal modes (displacement vector).

Table S2. Simulated vibrational frequencies of the tungsten complex **1** in cm^{-1} obtained at the B3LYP/def2-SVP level of theory.

19	124	280	569	869	1026	1334	1419	1701	3002
25	135	294	575	931	1032	1344	1422	1885	3008
32	140	296	611	933	1047	1350	1423	2534	3009
36	143	323	620	934	1069	1351	1423	2927	3012
39	146	328	628	938	1071	1351	1424	2930	3016
51	155	333	639	959	1075	1352	1429	2931	3023
58	167	337	656	962	1083	1353	1430	2931	3024
60	171	340	658	970	1124	1359	1431	2938	3028
66	178	368	661	973	1131	1362	1432	2940	3029
68	185	372	678	977	1156	1362	1433	2941	3036
71	191	384	680	984	1161	1364	1435	2941	3037
74	207	396	696	997	1168	1397	1438	2943	3069
83	212	453	714	998	1171	1401	1446	2945	3071
85	215	478	724	998	1177	1405	1453	2971	3090
89	224	499	725	999	1225	1406	1478	2976	3105
93	225	518	726	1011	1230	1408	1486	2978	
101	229	520	730	1013	1238	1410	1489	2985	
106	231	522	765	1013	1240	1411	1534	2989	
110	242	555	822	1022	1255	1412	1536	2990	
112	263	562	827	1025	1281	1415	1538	2993	
116	271	562	829	1025	1319	1417	1546	2996	
122	274	566	854	1026	1326	1419	1556	3001	

Table S3. Simulated Franck-Condon physics of [**2**]$^+$ as obtained by the B3LYP functional within the equilibrated singlet ground state structure (S_0). Prominent dipole-allowed singlet-singlet transitions contributing the UV-vis absorption (left) and spin-forbidden singlet-triplet transitions (right).

Excitation $S_0 \rightarrow S_i$	Character	ΔE [eV]	λ [nm]	f	Excitation $S_0 \rightarrow T_i$	Character	ΔE [eV]	λ [nm]	f
S_1	$MC_W/MLCT_W$	1.72	719	0.0140	T_1	$MC_W/MLCT_W$	1.27	974	-
S_2	$MLCT_{bpy}$	1.88	658	0.0116	T_2	$MLCT_{bpy}$	1.70	728	-
S_7	$MLCT_{bpy}$/MMCT	2.31	536	0.0401	T_3	$MLCT_{bpy}$	1.80	689	-
S_8	$MLCT_{bpy}$	2.37	523	0.0415	T_4	$MLCT_{bpy}$	1.90	654	-
S_9	$MLCT_{bpy}$	2.43	510	0.0748	T_5	$MLCT_{bpy}$	2.00	619	-
S_{10}	$MLCT_{bpy}$/MMCT	2.55	486	0.0788	T_6	$MLCT_{bpy}$	2.04	608	-
S_{13}	$MLCT_{bpy}$	2.74	453	0.0294	T_7	$MLCT_{bpy}$	2.10	591	-
S_{14}	$MLCT_{bpy}$/MMCT	2.81	441	0.0259	T_8	$MLCT_{bpy}$	2.18	567	-
S_{19}	$MLCT_{bpy}$	3.09	402	0.0365	T_9	$MLCT_{bpy}$	2.20	563	-
S_{24}	$MLCT_{bpy}$	3.20	388	0.0335	T_{10}	$MLCT_{bpy}$	2.32	535	-
S_{28}	$MLCT_{bpy}$	3.28	378	0.0539	T_{11}	$MLCT_{bpy}$	2.44	507	-
S_{33}	$MLCT_{bpy}$	3.41	363	0.0684	T_{12}	$MLCT_{bpy}$	2.51	493	-
S_{81}	LLCT	4.41	281	0.1715	T_{13}	$MLCT_{bpy}$/MMCT	2.64	470	-
S_{86}	LLCT	4.48	276	0.1654	T_{14}	$MLCT_{bpy}$/MMCT	2.68	463	-
S_{87}	LLCT	4.49	276	0.2619	T_{15}	$MLCT_{bpy}$/MMCT	2.82	439	-
					T_{16}	$MLCT_{bpy}$	2.87	431	-
					T_{17}	$MLCT_{bpy}$	2.88	430	-
					T_{18}	$MLCT_{bpy}$	2.89	430	-
					T_{19}	$MLCT_{bpy}$	2.93	423	-
					T_{20}	$MLCT_{bpy}$	2.98	416	-

Table S4. Electronic characters – as visualized by charge density differences (CDDs) – of prominent singlet-singlet ($S_0 \rightarrow S_i$) and singlet-triplet ($S_0 \rightarrow T_i$) excitations within the Franck-Condon geometry (i.e., S_0 equilibrium) of [**2**]$^+$ as obtained by the B3LYP functional. Charge transfer takes place from red to blue.

singlet-singlet ($S_0 \rightarrow S_i$) excitations

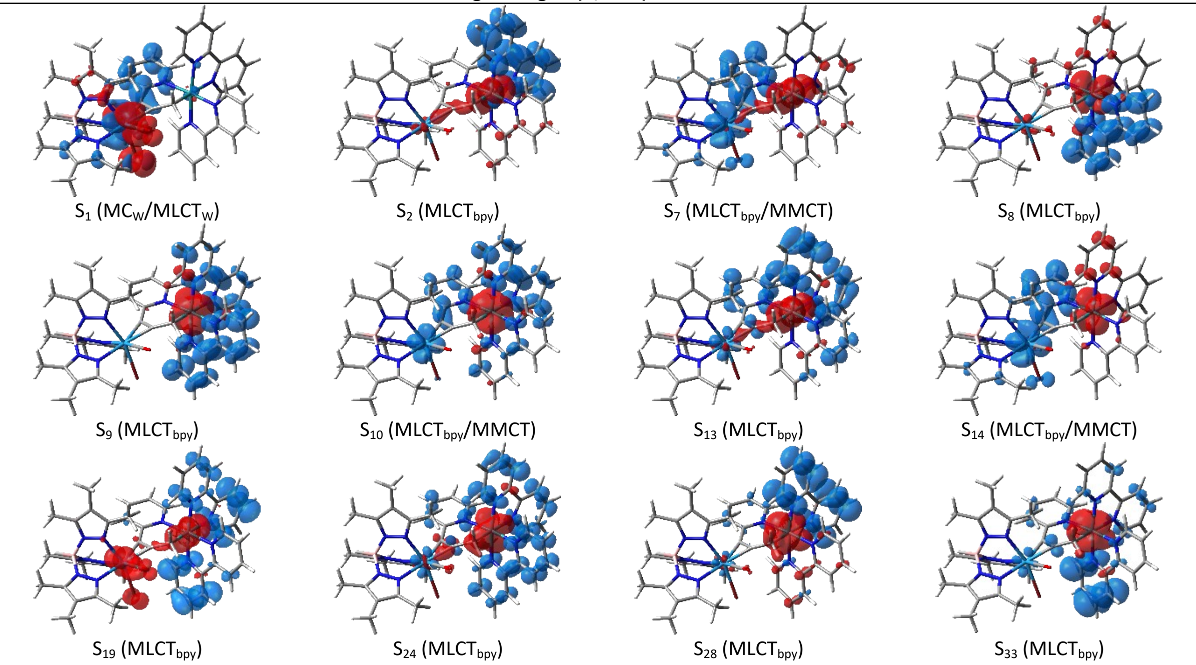

S_1 ($MC_W/MLCT_W$) | S_2 ($MLCT_{bpy}$) | S_7 ($MLCT_{bpy}$/MMCT) | S_8 ($MLCT_{bpy}$)

S_9 ($MLCT_{bpy}$) | S_{10} ($MLCT_{bpy}$/MMCT) | S_{13} ($MLCT_{bpy}$) | S_{14} ($MLCT_{bpy}$/MMCT)

S_{19} ($MLCT_{bpy}$) | S_{24} ($MLCT_{bpy}$) | S_{28} ($MLCT_{bpy}$) | S_{33} ($MLCT_{bpy}$)

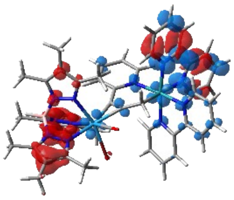
S_{81} (LLCT)

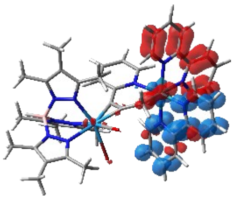
S_{86} (LLCT)

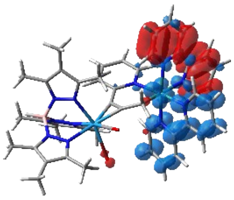
S_{87} (LLCT)

singlet-triplet ($S_0 \rightarrow T_i$) excitations

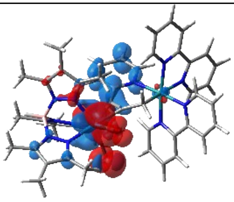
T_1 (MC_W/$MLCT_W$)

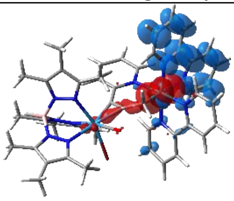
T_2 ($MLCT_{bpy}$)

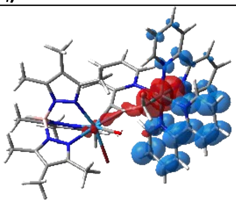
T_3 ($MLCT_{bpy}$)

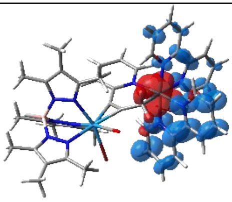
T_4 ($MLCT_{bpy}$)

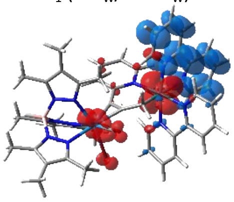
T_5 ($MLCT_{bpy}$)

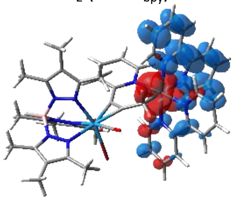
T_6 ($MLCT_{bpy}$)

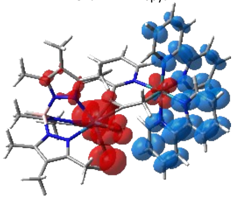
T_7 ($MLCT_{bpy}$)

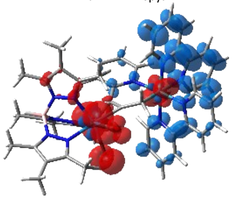
T_8 ($MLCT_{bpy}$)

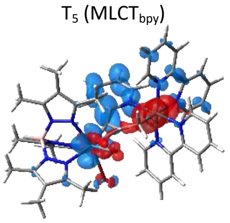
T_9 ($MLCT_{bpy}$)

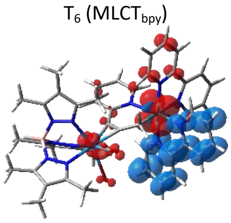
T_{10} ($MLCT_{bpy}$)

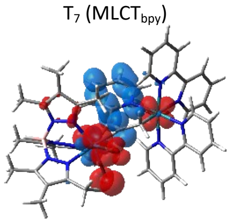
T_{11} ($MLCT_{bpy}$)

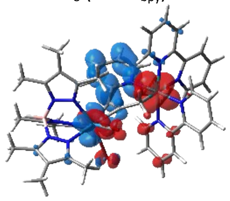
T_{12} ($MLCT_{bpy}$)

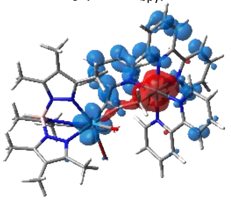
T_{13} ($MLCT_{bpy}$/MMCT)

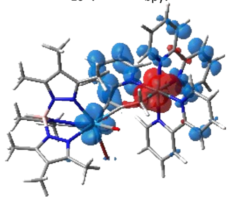
T_{14} ($MLCT_{bpy}$/MMCT)

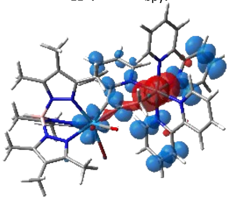
T_{15} ($MLCT_{bpy}$/MMCT)

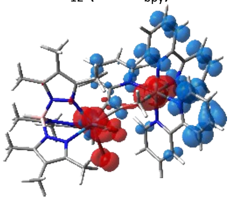
T_{16} ($MLCT_{bpy}$)

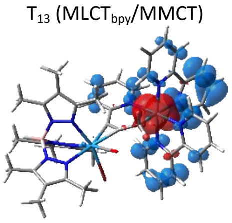
T_{17} ($MLCT_{bpy}$)

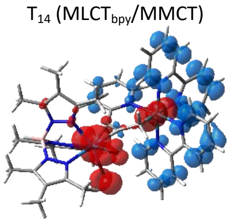
T_{18} ($MLCT_{bpy}$)

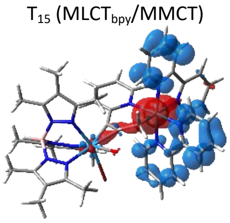
T_{19} ($MLCT_{bpy}$)

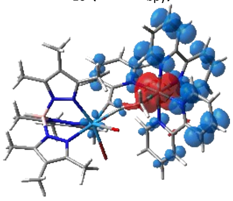
T_{20} ($MLCT_{bpy}$)

Table S5. Spin-orbit coupling elements ($\langle T_j | \hat{H}_{SOC} | S_i \rangle$ in cm^{-1}) between prominent excited singlet and triplet states of [**2**]$^+$. Excitation energies (E in eV) are given for all singlet and triplet excitations; oscillator strengths (f) are provided for singlet-singlet excitations. The provided triplet states are selected to match the energy level of the highest energy and dipole-allowed singlet transition (i.e. into S_{11}). All results were obtained by TD-B3LYP as implemented in Orca 5.0. Large spin-orbit coupling elements between S_0 and T_1 as well as between S_{10} and S_{11} and the respective triplet states are highlighted.

		S_0	S_5	S_7	S_8	S_9	S_{10}	S_{11}	S_{17}	S_{18}
	f	-	0.0420	0.0272	0.0319	0.0458	0.0678	0.0605	0.0342	0.0158
	ΔE [eV]	0.00	2.16	2.30	2.37	2.42	2.43	2.57	2.96	2.98
T_1	0.98	**1440**	341	27	69	68	**123**	**108**	385	401
T_2	1.73	232	98	236	98	83	**151**	**184**	75	40
T_3	1.82	147	57	214	208	263	**119**	**119**	32	18
T_4	1.89	106	78	183	103	137	88	**114**	32	62
T_5	2.00	388	60	101	113	67	**170**	**151**	156	237
T_6	2.02	415	90	71	108	73	**189**	**177**	195	266
T_7	2.04	99	20	28	67	262	**176**	27	31	30
T_8	2.22	128	30	114	122	98	61	98	6	20
T_9	2.30	186	125	74	91	19	74	**140**	37	61
T_{10}	2.33	193	298	40	66	54	55	**170**	74	214
T_{11}	2.36	102	31	63	87	57	39	50	33	20
T_{12}	2.46	397	230	17	145	77	**248**	**98**	187	552
T_{13}	2.54	328	135	21	36	17	48	**102**	160	658
T_{14}	2.60	441	144	31	76	40	**129**	**92**	110	183
T_{15}	2.68	347	240	20	8	13	48	**101**	721	1630
T_{16}	2.72	610	165	6	80	45	109	85	431	510
T_{17}	2.81	228	213	29	79	49	95	74	1214	2024
T_{18}	2.84	391	101	10	56	51	92	38	394	579
T_{19}	2.87	242	62	19	58	29	52	58	88	190
T_{20}	2.90	165	68	59	43	37	22	38	101	170

Table S6. Intersystem crossing rates (k_{ISC} in s^{-1}) between prominent excited singlet and triplet states of [**2**]$^+$. The provided triplet states are selected to match the energy level of the highest energy and dipole-allowed singlet transition (i.e. into S_{11}). All results were obtained by TD-B3LYP as implemented in Orca 5.0. Large spin-orbit coupling elements between S_0 and T_1 as well as between S_{10} and S_{11} and the respective triplet states are highlighted.

		S_5	S_7	S_8	S_9	S_{10}	S_{11}	S_{17}	S_{18}
	f	0.0420	0.0272	0.0319	0.0458	0.0678	0.0605	0.0342	0.0158
	Δ*E* [eV]	2.16	2.30	2.37	2.42	2.43	2.57	2.96	2.98
T_1	0.98	3.92×10^{11}	1.99×10^{9}	1.15×10^{10}	1.03×10^{10}	3.35×10^{10}	2.17×10^{10}	1.76×10^{11}	1.88×10^{11}
T_2	1.73	2.27×10^{11}	7.67×10^{11}	1.06×10^{11}	6.61×10^{10}	2.11×10^{11}	2.18×10^{11}	1.72×10^{10}	4.72×10^{9}
T_3	1.82	1.20×10^{11}	8.88×10^{11}	6.53×10^{11}	8.70×10^{11}	1.73×10^{11}	1.13×10^{11}	3.70×10^{9}	1.05×10^{9}
T_4	1.89	3.61×10^{11}	8.99×10^{11}	2.12×10^{11}	3.05×10^{11}	1.22×10^{11}	1.29×10^{11}	4.08×10^{9}	1.50×10^{10}
T_5	2.00	4.87×10^{11}	4.88×10^{11}	4.19×10^{11}	1.14×10^{11}	7.01×10^{11}	3.15×10^{11}	1.22×10^{11}	2.71×10^{11}
T_6	2.02	1.26×10^{12}	2.64×10^{11}	4.08×10^{11}	1.44×10^{11}	9.30×10^{11}	4.55×10^{11}	1.95×10^{11}	3.52×10^{11}
T_7	2.04	8.13×10^{10}	4.90×10^{10}	1.83×10^{11}	2.11×10^{12}	9.07×10^{11}	1.18×10^{10}	5.24×10^{9}	4.63×10^{9}
T_8	2.22	3.07×10^{10}	3.62×10^{12}	2.17×10^{12}	8.84×10^{11}	3.14×10^{11}	3.28×10^{11}	3.24×10^{8}	3.04×10^{9}
T_9	2.30	1.17×10^{10}	2.57×10^{12}	2.55×10^{12}	6.81×10^{10}	9.21×10^{11}	1.05×10^{12}	1.36×10^{10}	3.62×10^{10}
T_{10}	2.33	1.07×10^{10}	1.76×10^{11}	1.85×10^{12}	7.85×10^{11}	7.51×10^{11}	1.97×10^{12}	6.34×10^{10}	5.00×10^{11}
T_{11}	2.36	3.34×10^{7}	1.31×10^{11}	3.51×10^{12}	1.10×10^{12}	4.71×10^{11}	2.04×10^{11}	1.34×10^{10}	4.73×10^{9}
T_{12}	2.46	1.91×10^{7}	7.75×10^{7}	1.42×10^{11}	5.08×10^{11}	7.86×10^{12}	1.94×10^{12}	6.17×10^{11}	5.05×10^{12}
T_{13}	2.54	2.00×10^{5}	2.78×10^{6}	1.87×10^{8}	5.13×10^{8}	6.82×10^{9}	4.27×10^{12}	6.28×10^{11}	9.85×10^{12}
T_{14}	2.60	1.66×10^{4}	4.01×10^{5}	5.10×10^{7}	1.66×10^{8}	2.66×10^{9}	1.46×10^{12}	3.94×10^{11}	1.01×10^{12}
T_{15}	2.68	1.49×10^{3}	4.74×10^{3}	1.45×10^{4}	4.34×10^{5}	8.85×10^{6}	3.99×10^{10}	2.65×10^{13}	1.23×10^{14}
T_{16}	2.72	1.04×10^{2}	6.31×10^{1}	2.00×10^{5}	6.83×10^{5}	5.95×10^{6}	3.14×10^{9}	1.27×10^{13}	1.60×10^{13}
T_{17}	2.81	3.77	2.82×10^{1}	3.69×10^{3}	1.40×10^{4}	7.94×10^{4}	3.29×10^{7}	2.12×10^{14}	5.10×10^{14}
T_{18}	2.84	2.94×1^{-1}	1.17	6.38×10^{2}	5.20×10^{3}	2.50×10^{4}	2.72×10^{6}	2.82×10^{13}	5.24×10^{13}
T_{19}	2.87	3.84×10^{-2}	1.380	2.20×10^{2}	5.52×10^{2}	2.59×10^{3}	2.01×10^{6}	1.81×10^{12}	7.24×10^{12}
T_{20}	2.90	1.15×10^{-2}	3.29	2.97×10^{1}	2.11×10^{2}	1.12×10^{2}	1.95×10^{5}	3.33×10^{12}	8.08×10^{12}

Table S7. Intersystem crossing times (τ_{ISC} in ps) between prominent excited singlet and triplet states of [**2**]$^+$. The provided triplet states are selected to match the energy level of the highest energy and dipole-allowed singlet transition (i.e. into S_{11}). All results were obtained by TD-B3LYP as implemented in Orca 5.0. Sub-ps processes are highlighted.

		S_5	S_7	S_8	S_9	S_{10}	S_{11}	S_{17}	S_{18}
	f	0.0420	0.0272	0.0319	0.0458	0.0678	0.0605	0.0342	0.0158
	Δ*E* [eV]	2.16	2.30	2.37	2.42	2.43	2.57	2.96	2.98
T_1	0.98	2.55	5.01×10^{2}	8.73×10	9.68×10	2.99×10	4.62×10	5.69	5.31
T_2	1.73	4.40	1.30	9.39	1.51×10	4.75	4.60	5.83×10	2.12×10^{2}
T_3	1.82	8.31	1.13	1.53	1.15	5.78E	8.84	2.70×10^{2}	9.48×10^{2}
T_4	1.89	2.77	1.11	4.72	3.28	8.17	7.76	2.45×10^{2}	6.68×10
T_5	2.00	2.05	2.05	2.39	8.78	1.43	3.18	8.18	3.68
T_6	2.02	**7.96×10^{-1}**	3.79	2.45	6.96	1.08	2.20	5.13	2.84
T_7	2.04	1.23×10	2.04×10	5.46	**4.75×10^{-1}**	1.10	8.45×10	1.91×10^{2}	2.16×10^{2}
T_8	2.22	3.26×10	**2.76×10^{-1}**	**4.60×10^{-1}**	1.13	3.18	3.05	3.09×10^{3}	3.29×10^{2}
T_9	2.30	8.54×10	**3.89×10^{-1}**	**3.92×10^{-1}**	1.47×10	1.09	**9.54×10^{-1}**	7.33×10	2.76×10
T_{10}	2.33	9.34×10	5.68	**5.40×10^{-1}**	1.27	1.33	**5.07×10^{-1}**	1.58×10	2.00
T_{11}	2.36	2.99×10^{4}	7.61	**2.85×10^{-1}**	**9.11×10^{-1}**	2.12	4.90	7.45×10^{1}	2.11×10^{2}
T_{12}	2.46	5.23×10^{4}	1.29×10^{4}	7.07	1.97	**1.27×10^{-1}**	**5.16×10^{-1}**	1.62	**1.98×10^{-1}**
T_{13}	2.54	5.00×10^{6}	3.59×10^{4}	5.34×10^{3}	1.95×10^{3}	1.47×10^{2}	**2.34×10^{-1}**	1.59	**1.02×10^{-1}**
T_{14}	2.60	6.02×10^{7}	2.50×10^{6}	1.96×10^{4}	6.02×10^{3}	3.76×10^{2}	**6.84×10^{-1}**	2.54	**9.95×10^{-1}**
T_{15}	2.68	6.73×10^{8}	2.11×10^{8}	6.90×10^{7}	2.30×10^{6}	1.13×10^{5}	2.51×10	**3.78×10^{-2}**	**8.16×10^{-3}**
T_{16}	2.72	9.60×10^{9}	1.59×10^{10}	5.00×10^{6}	1.46×10^{6}	1.68×10^{5}	3.18×10^{2}	**7.86×10^{-2}**	**6.26×10^{-2}**
T_{17}	2.81	2.65×10^{11}	3.55×10^{10}	2.71×10^{8}	7.13×10^{7}	1.26×10^{7}	3.04×10^{4}	**4.72×10^{-3}**	**1.96×10^{-3}**
T_{18}	2.84	3.40×10^{12}	8.53×10^{11}	1.57×10^{9}	1.92×10^{8}	4.00×10^{7}	3.68×10^{5}	**3.54×10^{-2}**	**1.91×10^{-2}**
T_{19}	2.87	2.61×10^{13}	7.24×10^{11}	4.54×10^{9}	1.81×10^{9}	3.87×10^{8}	4.97×10^{5}	**5.53×10^{-1}**	**1.38×10^{-1}**
T_{20}	2.90	8.72×10^{13}	3.04×10^{11}	3.37×10^{10}	4.74×10^{9}	8.93×10^{9}	5.14×10^{6}	**3.01×10^{-1}**	**1.24×10^{-1}**

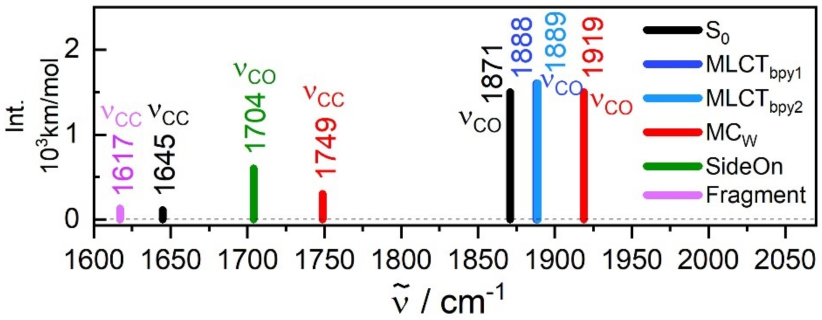

Figure S4. Simulated IR spectra of the W-Ru complex [**2**]$^+$ obtained at the B3LYP/def2-SVP level of theory for the ground state at the Franck-Condon point and the fully relaxed $^3MLCT_{bpy2}$ (light blue), $^3MLCT_{bpy1}$ (dark blue), ^3MC_W (red), the Side-on species (green), and the fragment after CO abstraction (pink).

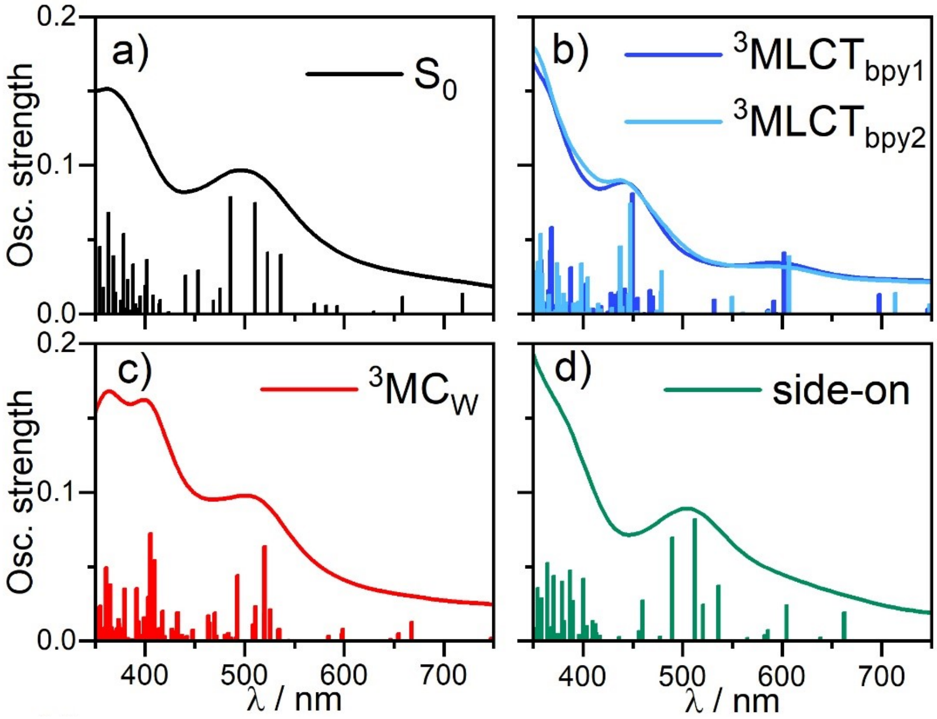

Figure S5. Simulated electronic absorption spectra for the W-Ru complex [**2**]$^+$ obtained at the B3LYP/def2-SVP level of theory given by dipole-allowed singlet-singlet transitions for the ground state at the Franck-Condon point (a) and the *side-on* geometry (d), as well as triplet-triplet transitions for the fully relaxed $^3MLCT_{bpy2}$/$^3MLCT_{bpy1}$ (b) and ^3MC_W (c) states. The absorption spectra in (a)-(d) were obtained by convolution of the calculated stick spectra with a Lorentzian line shape function of 0.4 eV width (FWHM).

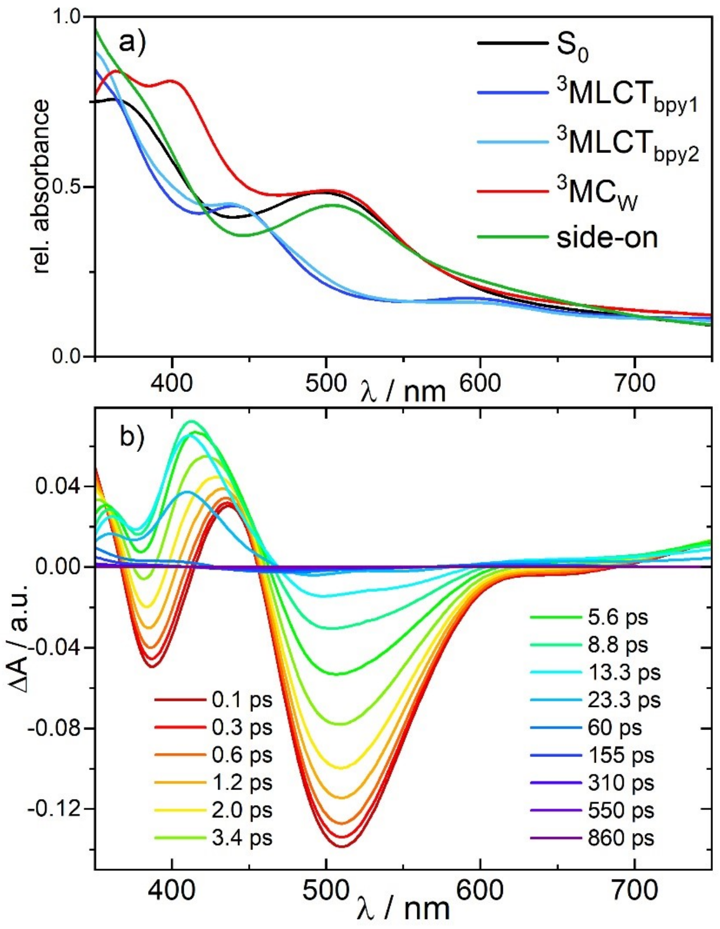

Figure S6. (a) Comparison of the simulated electronic absorption spectra for the W-Ru complex [**2**]$^+$ from Figure S5. (b) Simulated temporal evolution of the UV-vis transients based on the kinetic model presented in Figure 7 of the main paper.

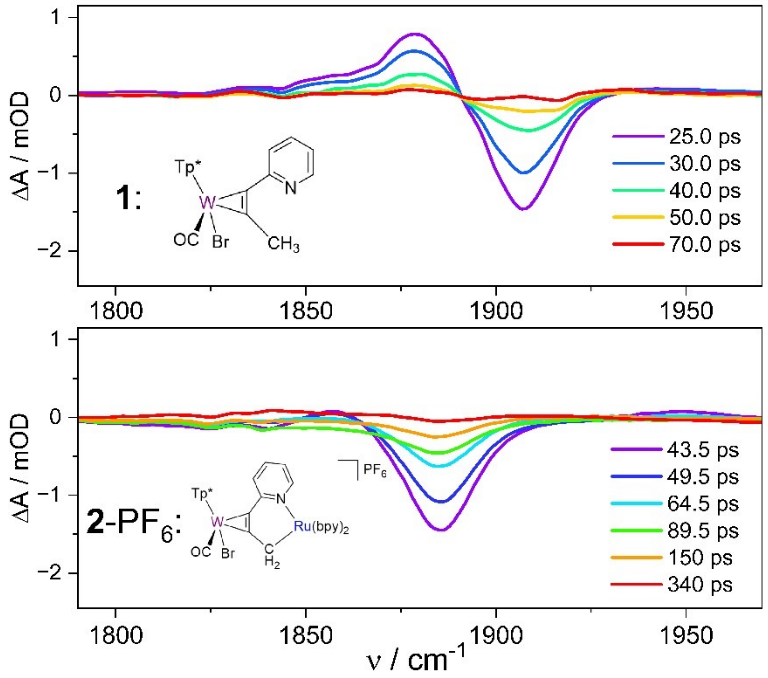

Figure S7. Transient IR spectra for **1** (upper panel) and **2**-PF_6 (lower panel) at large pump-probe delays. During the recovery of the ground state of **1**, the bleaching of the v = 0→1 transition is balanced by red-shifted positive absorptions from excited CO vibrational states. For **2**-PF_6, these excited state bands are absent during the lifetime of I.

Table S8. Simulated excited state properties contributing to the excited-state absorption of [**2**]$^+$ as obtained by B3LYP/def2-SVP within the equilibrated ^{3}MLCT$_{bpy2}$, ^{3}MLCT$_{bpy1}$, ^{3}MC$_W$ and ^{1}*side-on*. Prominent dipole-allowed triplet-triplet (^{3}MLCT$_{bpy2}$, ^{3}MLCT$_{bpy1}$, ^{3}MC$_W$) and singlet-singlet transitions (^{1}*side-on*) are indicated.

**^{3}MLCT$_{bpy2}$ (1.54 eV), triplet-triplet ($T_1 \rightarrow T_i$) excitations**				
Excitation $T_1 \rightarrow T_i$	Character	ΔE [eV]	λ [nm]	f
T_{11}	IL$_{bpy}$	1.50	826	0.0161
T_{14}	LMCT$_{bpy}$	2.04	607	0.0391
T_{22}	MLCT$_{bpy}$	2.59	479	0.0293
T_{29}	IL$_{bpy}$	2.77	447	0.0745
T_{32}	MLCT$_{bpy}$	2.83	437	0.0456
T_{40}	MLCT$_{bpy}$	3.06	405	0.0247

**relaxed ^{3}MLCT$_{bpy1}$ (1.32 eV)**				
Excitation $T_1 \rightarrow T_i$	Character	ΔE [eV]	λ [nm]	f
T_{11}	IL$_{bpy}$	1.56	794	0.0231
T_{14}	LMCT$_{bpy}$	2.06	602	0.0413
T_{24}	MLCT$_{bpy}$	2.65	467	0.0161
T_{27}	IL$_{bpy}$	2.76	450	0.0812
T_{40}	MLCT$_{bpy}$	3.08	403	0.0203

**^{3}MC$_W$ (0.82 eV), triplet-triplet ($T_1 \rightarrow T_i$) excitations**				
Excitation $T_1 \rightarrow T_i$	Character	ΔE [eV]	λ [nm]	f
T_4	MLCT$_W$	1.62	764	0.0239
T_{22}	MLCT$_{bpy}$	2.38	520	0.0637
T_{23}	LMCT$_W$	2.39	520	0.0192
T_{24}	MLCT$_{bpy}$	2.43	511	0.0235
T_{26}	MLCT$_{bpy}$	2.52	493	0.0443
T_{53}	MC$_W$	3.03	409	0.0541
T_{55}	MLCT$_{bpy}$	3.06	405	0.0726

**^{1}Side-on (1.83 eV), triplet-triplet ($S_0 \rightarrow S_i$) excitations**				
Excitation $S_0 \rightarrow S_i$	Character	ΔE [eV]	λ [nm]	f
S_2	MLCT$_{bpy}$	1.87	662	0.0194
S_4	MLCT$_{bpy}$/MMCT	2.05	604	0.0242
S_8	MLCT$_{bpy}$/MMCT	2.31	536	0.0375
S_{10}	MLCT$_{bpy}$	2.42	512	0.0822
S_{11}	MLCT$_{bpy}$/MMCT	2.53	489	0.0696
S_{13}	MLCT$_{bpy}$	2.70	460	0.0276

Table S9. Electronic characters – as visualized by charge density differences (CDDs) – of prominent triplet-triplet ($T_1 \rightarrow T_i$) transitions (^{3}MLCT$_{bpy2}$, ^{3}MLCT$_{bpy1}$ and ^{3}MC$_W$) and singlet-singlet ($S_0 \rightarrow S_i$) excitations within the fully equilibrated photo-intermediates of [**2**]$^+$ as obtained by the B3LYP functional. Charge transfer takes place from red to blue.

^{3}MLCT$_{bpy2}$ (1.54 eV), triplet-triplet ($T_1 \rightarrow T_i$) excitations

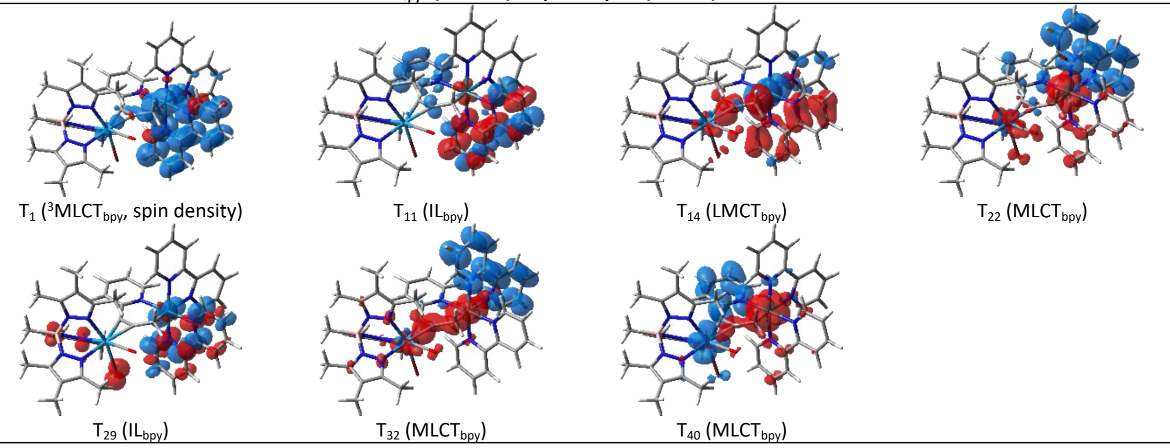

T_1 (^{3}MLCT$_{bpy}$, spin density) | T_{11} (IL$_{bpy}$) | T_{14} (LMCT$_{bpy}$) | T_{22} (MLCT$_{bpy}$) | T_{29} (IL$_{bpy}$) | T_{32} (MLCT$_{bpy}$) | T_{40} (MLCT$_{bpy}$)

^{3}MLCT$_{bpy1}$ (1.32 eV), triplet-triplet ($T_1 \rightarrow T_i$) excitations

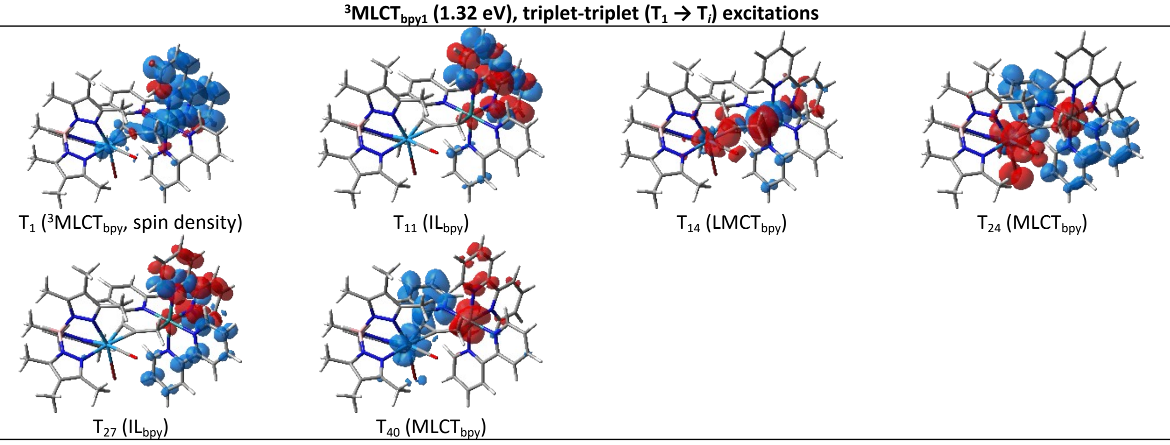

T_1 (^{3}MLCT$_{bpy}$, spin density) | T_{11} (IL$_{bpy}$) | T_{14} (LMCT$_{bpy}$) | T_{24} (MLCT$_{bpy}$) | T_{27} (IL$_{bpy}$) | T_{40} (MLCT$_{bpy}$)

^{3}MC$_W$ (0.82 eV), triplet-triplet (T$_1$ → T$_i$) excitations

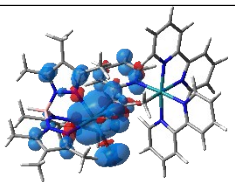

T$_1$ (^{3}MC$_W$, spin density)

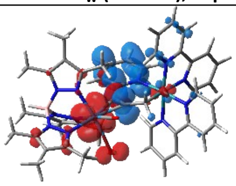

T$_4$ (MLCT$_W$)

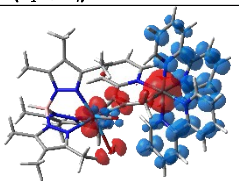

T$_{22}$ (MLCT$_{bpy}$)

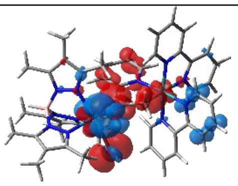

T$_{23}$ (LMCT$_W$)

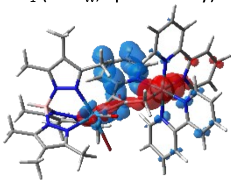

T$_{23}$ (MLCT$_{bpy}$)

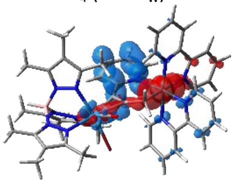

T$_{24}$ (MLCT$_{bpy}$)

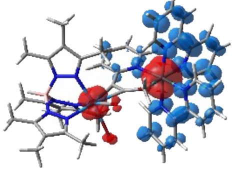

T$_{26}$ (MLCT$_{bpy}$)

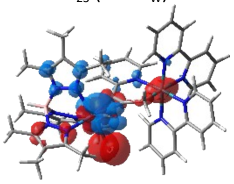

T$_{53}$ (MC$_W$)

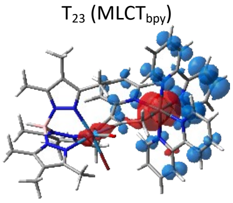

T$_{55}$ (MLCT$_{bpy}$)

^{1}*side-on* (1.83 eV), triplet-triplet (S$_0$ → S$_i$) excitations

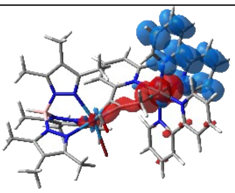

S$_2$ (MLCT$_{bpy}$)

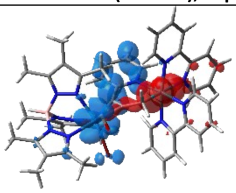

S$_4$ (MLCT$_{bpy}$/MMCT)

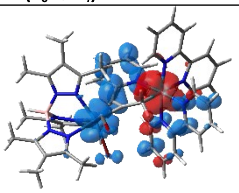

S$_8$ (MLCT$_{bpy}$/MMCT)

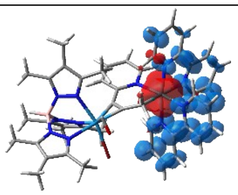

S$_{10}$ (MLCT$_{bpy}$)

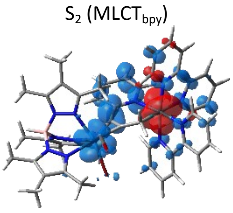

S$_{11}$ (MLCT$_{bpy}$/MMCT)

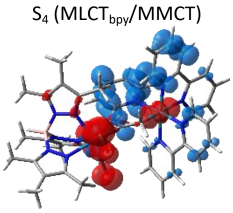

S$_{13}$ (MLCT$_{bpy}$)

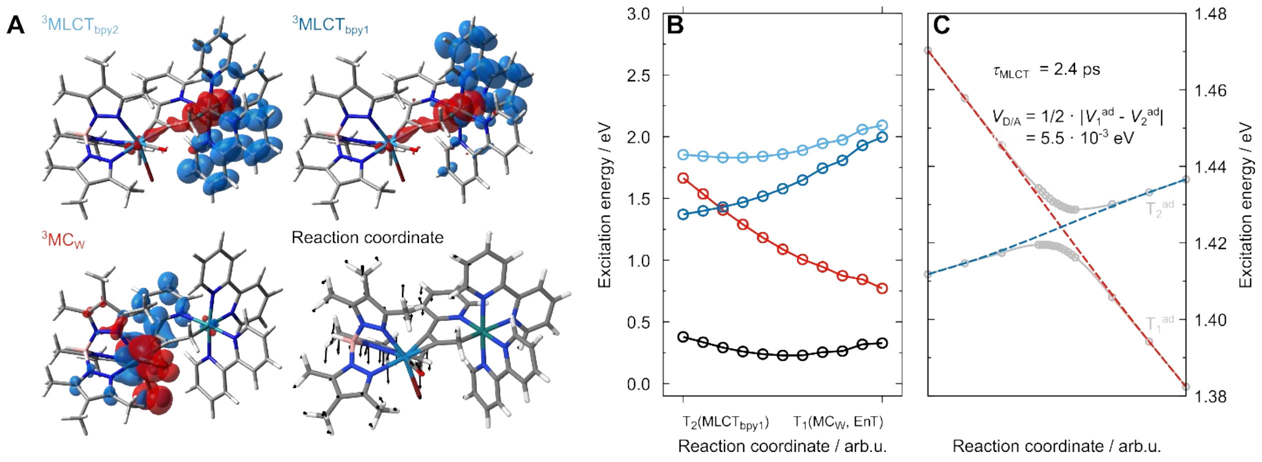

Figure S8. A: Electronic characters of triplet states involved in the ^{3}MLCT$_{bpy}$-^{3}MC$_W$ population transfer of [**2**]$^+$ as visualized by charge density difference plots (charge transfer occurs from red to blue) and linear-interpolated internal coordinate (LIIC) connecting the fully equilibrated ^{3}MLCT$_{bpy1}$ and ^{3}MC$_W$ structures. **B:** Potential energy curves of the respective electronic states (in addition S_0, in black) along the LIIC. **C:** Zoomed in crossing region between the diabatic states of interest, i.e. the ^{3}MLCT$_{bpy1}$ donor state and the ^{3}MC$_W$ acceptor state; adiabatic energies (T_1 and T_2) are shown in grey. Simulated lifetime obtained as $(k)^{-1}$ (see S18) and potential coupling $V_{D/A}$ as (see S20) are provided as inset. Diabatization was performed by following the electronic character of the states of interest.

3. References

1 J. Franz, M. Oelschlegel, J. P. Zobel, S.-A. Hua, J.-H. Borter, L. Schmid, G. Morselli, O. S. Wenger, D. Schwarzer, F. Meyer, and L. González *J. Am. Chem. Soc.* 2024, **146**, 11272.

2 M. J. Frisch, G. W. Trucks, H. B. Schlegel, G. E. Scuseria, M. A. Robb, J. R. Cheeseman, G. Scalmani, V. Barone, G. A. Petersson, H. Nakatsuji,X. Li, M. Caricato, A. V. Marenich, J. Bloino, B. G. Janesko, R. Gomperts, B. Mennucci, H. P. Hratchian, J. V. Ortiz, A. F. Izmaylov, J. L. Sonnenberg, D. Williams-Young, F. Ding, F. Lipparini, F. Egidi, J. Goings, B. Peng, A. Petrone, T. Henderson, D. Ranasinghe, V. G. Zakrzewski, J. Gao, N. Rega, G. Zheng, W. Liang, M. Hada, M. Ehara, K. Toyota, R. Fukuda, J. Hasegawa, M. Ishida, T. Nakajima, Y. Honda, O. Kitao, H. Nakai, T. Vreven, K. Throssell, J. A. Montgomery, Jr., J. E. Peralta, F. Ogliaro, M. J. Bearpark, J. J. Heyd, E. N. Brothers, K. N. Kudin, V. N. Staroverov, T. A. Keith, R. Kobayashi, J.Normand, K. Raghavachari, A. P. Rendell, J. C. Burant, S. S. Iyengar, J. Tomasi, M. Cossi, J. M. Millam, M. Klene, C. Adamo, R. Cammi, J. W. Ochterski, R. L. Martin, K. Morokuma, O. Farkas, J. B. Foresman, D. J. Fox, *Gaussian 16*; Gaussian, Inc., Wallingford CT, 2016.

3 a) A. D. Becke *J. Chem. Phys.*1993, **98**, 5648; b) A. D. Becke, *Phys. Rev. A Gen. Phys.* 1988, **38**, 3098; c) C. Lee, W. Yang, R. G. Parr, *Phys. Rev. B, Condens. Matter* 1988, **37**, 785.

4 a) F. Weigend, R. Ahlrichs, *Phys. Chem. Chem. Phys.* 2005, **7**, 2663933; b) F. Weigend, *Phys. Chem. Chem. Phys.* 2006, **8**, 1057.

5 S. Grimme, S. Ehrlich, L. Goerigk, *J. Comput. Chem.* 2011, **32**, 1456.

6 a) A. V. Marenich, C. J. Cramer, D. G. Truhlar, *J. Phys. Chem. B* 2009, **113**, 6378; b) B. Mennucci, C. Cappelli, C. A. Guido, R. Cammi, J. Tomasi, *J. Phys. Chem. A* 2009, **113**, 3009.

7 a) J. P. Merrick, D. Moran, L. Radom, *J. Phys. Chem. A* 2007, **111**, 11683; b) https://cccbdb.nist.gov/vibscalejustx.asp.

8 S. Kupfer, Zenodo, 2025, doi.org/10.5281/zenodo.15083786.

9 a) L. Zedler, A. K. Mengele, K. M. Ziems, Y. Zhang, M. Wächtler, S. Gräfe, T. Pascher, S. Rau, S. Kupfer, B. Dietzek, *Angew. Chem. Int. Ed.* 2019, **58**, 13140; b) G. Yang, G. E. Shillito, C. Zens, B. Dietzek-Ivanšić, S. Kupfer, *J. Chem. Phys.* 2023, **159**, 024109; c) G. E. Shillito, S. Rau, S. Kupfer, *ChemCatChem* 2023, **15**, e202201489; d) L. Zedler, S. Kupfer, H. Schmidt, B. Dietzek-Ivanšić, *Chem. Eur. J.* 2024, **30**, e202303079; e) G. Yang, L. Blechschmidt, L. Zedler, C. Zens, K. Witas, M. Schmidt, B. Esser, S. Rau, G. E. Shillito, B. Dietzek-Ivanšić, S. Kupfer, Chem. *Eur. J.* 2025, **31**, e202404671.

10 F. Neese, F. Wennmohs, U. Becker, C. Riplinger, *J. Chem. Phys.* 2020, **152**, 224108.

11 K. Shizu, H. Kaji, *J. Phys. Chem. A* 2021, **125**, 9000.

12 G. Yang, G. E. Shillito, P. Seeber, O. S. Wenger and S. Kupfer, *Chem. Sci.* 2025, **16**, 18113.

13 a) A. Koch, D. Kinzel, F. Dröge, S. Gräfe, S. Kupfer, *J. Phys. Chem. C* 2017, **121**, 16066; b) M. Staniszewska, S. Kupfer, J. Guthmuller, *Chem. Eur J.* 2018, **24**, 11166; c) M. Staniszewska, S. Kupfer, J. Guthmuller, *J. Phys. Chem. C* 2019, **123**, 16003; d) C. Zens, C. Friebe, U. S. Schubert, M. Richter, S. Kupfer, *ChemSusChem.* 2023, **16**, e202201679.
